# Characterization of Temperature Gradients According to Height in a Baroque Church by Means of Wireless Sensors

**DOI:** 10.3390/s21206921

**Published:** 2021-10-19

**Authors:** Sandra Ramírez, Manuel Zarzo, Angel Perles, Fernando-Juan García-Diego

**Affiliations:** 1 Department of Applied Statistics, Operations Research and Quality, Universitat Politècnica de València, Camino de Vera s/n, 46022 Valencia, Spain; smramirez@javerianacali.edu.co (S.R.); mazarcas@eio.upv.es (M.Z.); 2 Department of Natural Sciences and Mathematics, Pontificia Universidad Javeriana Cali, Cali 760031, Colombia; 3 ITACA Institute, Universitat Politècnica de València, Camino de Vera s/n, 46022 Valencia, Spain; aperles@disca.upv.es; 4 Department of Applied Physics (U.D. Industrial Engineering), Universitat Politècnica de València, Camino de Vera s/n, 46022 Valencia, Spain

**Keywords:** autocorrelation, Holt-Winters, LDA, temperature gradient, sPLS, wireless sensors

## Abstract

The baroque church of Saint Thomas and Saint Philip Neri (Valencia, Spain), which was built between 1727 and 1736, contains valuable paintings by renowned Spanish artists. Due to the considerable height of the central nave, the church can experience vertical temperature gradients. In order to investigate this issue, temperatures were recorded between August 2017 and February 2018 from a wireless monitoring system composed of 21 sensor nodes, which were located at different heights in the church from 2 to 13 m from the floor level. For characterizing the temperature at high, medium and low altitude heights, a novel methodology is proposed based on sparse Partial Least Squares regression (sPLS), Linear Discriminant Analysis (LDA), and the Holt-Winters method, among others, which were applied to a time series of temperature. This approach is helpful to discriminate temperature profiles according to sensor height. Once the vertical thermal gradients for each month were characterized, it was found that temperature reached the maximum correlation with sensor height in the period between August 10th and September 9th. Furthermore, the most important features from the time series that explain this correlation are the mean temperature and the mean of moving range. In the period mentioned, the vertical thermal gradient was estimated to be about 0.043 ${}^{\circ }$C/m, which implies a difference of 0.47 ${}^{\circ }$C on average between sensor nodes at 2 m from the floor with respect to the upper ones located at 13 m from the floor level. The gradient was estimated as the slope from a linear regression model using height and hourly mean temperature as the predictor and response, respectively. This gradient is consistent with similar reported studies. The fact that such gradient was only found in one month suggests that the mechanisms of dust deposition on walls involved in vertical thermal gradients are not important in this case regarding the preventive conservation of artworks. Furthermore, the methodology proposed here was useful to discriminate the time series at high, medium and low altitude levels. This approach can be useful when a set of sensors is installed for microclimate monitoring in churches, cathedrals, and other historical buildings, at different levels and positions.

## 1. Introduction

Cultural heritage is a source of wealth because it promotes tourism, creative art and native culture. Tourists often select places to visit based on the culture and artistic significance of museums, monuments, exhibitions, and historical ruins, among other criteria. The protection and conservation of cultural heritage is a challenge because artworks undergo certain degradation over time. In order to prevent damage, artworks should be maintained in stable and controlled climatic scenarios. However, usually, such conditions are only achieved in museums [1].

Temperature (T) and relative humidity (RH) tend to be more stable inside a building, while outer air conditions present a higher daily and seasonal variability [2]. In fact, the most significant physical factors in the preservation of collections and artefacts are T and RH, which can potentially deteriorate or damage historical or cultural objects [3]. The requirements for appropriate control of indoor air conditions depend on the type of materials, some of which are very sensitive to sudden variations of T or RH. Thus, particular artworks demand specific microclimatic conditions. Furthermore, certain characteristics of buildings can generate more complex requirements because it is not possible to control the indoor environment [4,5,6,7]. The values of T and RH inside a historical building basically depend on the climatic conditions outside, apart from other factors such as construction materials, structure, and dimensions of the building. Variations in T and RH can induce thermal shock [2], air movements, wet–dry cycles [8,9], and surface or under-surface salt dissolution–crystallization [10]. Air movements, as well as wet and dry cycles are usually responsible for soiling processes and deterioration [11]. Furthermore, dissolution of alkaline surfaces can be caused by condensation that can be generated by either water vapour coming from open doors, human metabolism, or the use of lit candles. Furthermore, in the presence of high humidity and moderate temperatures, surface condensation and damp can give rise to biological colonization by insects, bacteria or fungi, which can generate biodeterioration in specific areas of the building [12]. In numerous cases, artworks in churches have been affected due to inappropriate microclimatic conditions [13].

In the Mediterranean region, the use of active air conditioning systems has been more common in modern spaces of worship, due to the growing request from the public for comfortable temperatures in the buildings. Such systems must guarantee conditions of wellbeing, safety and energy efficiency [14], but it is a challenge to satisfy at the same time the requirements of human comfort, the preservation of artworks and energy efficiency [14]. In most cases, such requisites cannot be fulfilled optimally. In Spain, as in other Mediterranean countries, the thermal conditions of historical buildings are not considered in current environmental conditioning regulations (e.g., European Standards EN 15757:2010 [15], which is based on laboratory tests [16] and on case studies [17,18,19].

**Figure 1:**
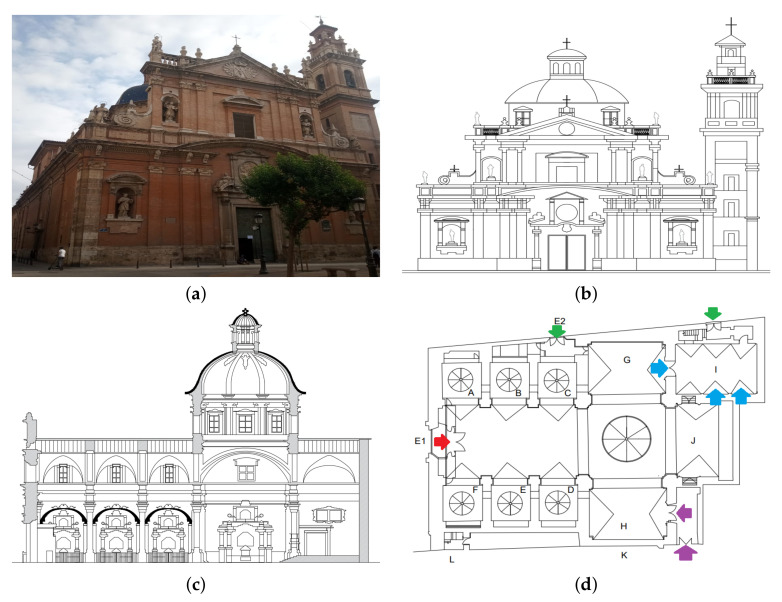
Church of Saint Thomas the Apostle and Saint Philip Neri in Valencia (Spain). (**a**) Front and side view of the church; (**b**) front view; (**c**) longitudinal section; (**d**) plan of the church, where the different observable structures are indicated: A. Baptismal chapel, B. Chapel of Our Lady of the Forsaken, C. Chapel of the Holy Trinity, D. Chapel of Our Lady of Mount Carmel, E. Chapel of the Calvary, F. Chapel of Saint Anthony of Padua, G. Altarpiece of Saint Joseph, H. Altarpiece of Our Lady of La Salette, I. Chapel of the Holy Communion, J. High altar and main altarpiece, K. Sacristy, L. Bell tower, E1. Main entrance, E2. Side entrance. The small circles indicate the projection of vaults of the internal chapels. The larger circle represents the projection of the main dome of the church. The arrows indicate the air inlet and sources of ventilation in the church.

In Spain, the majority of ancient churches, cathedrals and other historical buildings do not have air conditioning systems; as a consequence, artworks can experience harmful thermo-hygrometric oscillations, due to the outer climatic conditions. Moreover, these buildings are large, which favors vertical air flows that are related to the deposition of dust and dirt on walls, paintings, frescoes, altarpieces, and other artworks, which can require expensive cleaning and maintenance actions for an appropriate preservation of the cultural heritage. Vertical air movements can be caused by the ventilation, because many of these buildings have windows in the upper part, so the air enters through the main doors and lower inlets and leaves through the upper windows. The presence of vertical thermal gradients is another factor of vertical air movements, because hot air has a lower density and rises up. Therefore, studying the correlation between temperature and sensor height is of interest to assess the vertical air flows, which makes it possible to evaluate whether the gradient of T is acceptable or excessive, regarding the risk for dust deposition in walls and paintings. In case of inappropriate gradients, corrective actions might be proposed. The present research is intended to study vertical temperature gradients in the church of Saint Thomas and Saint Philip Neri in Valencia, Spain (Latitude: 39 30 N and Longitude: 000 28 W [20]), which has an unheated/natural microclimate indoor (see Figure 1). The climate in Valencia is classified as BsK (tropical and subtropical steppe) according to the Köppen classification [20]. The principal source of ventilation is through the main entrance of the church, and there are a few air inlets in the sacristy and the chapel of the Holy Communion though the former is separate from the main nave by a door that usually remains closed. This church contains valuable artworks, among these are paintings by renowned Spanish artists such as Juan de Juanes or Vicente Juan Macip (1507–1579), Jerónimo Jacinto de Espinosa (1600–1667), José Vergara (1726–1799), and Vicente López (1772–1850). These paintings are located in the main chapel, as well as the altarpieces of Saint Joseph and Our Lady of the Unforsaken (see Figure 1c,d).

### 1.1. Microclimatic Monitoring for the Preservation of Cultural Heritage

In recent years, European governments have funded different initiatives with the goal of preserving artworks in museums and similar buildings. For example, the CollectionCare Project is working at present on an innovative system of wireless sensors for the preservation of cultural heritage [21]. In this context, experts suggest that it is necessary to implement continuous monitoring systems to identify harmful microclimatic conditions that affect the works of art [22]. Long-term monitoring of indoor air conditions is a key issue according to the new requirements for preventive conservation [23]. Such systems require maintenance and routine practices [24]. Furthermore, practical solutions need to be proposed for the adaptation of climate change [25]. Furthermore, it is important to define the compatibility between the climate control potentials and the preservation requirements [22].

Many studies about the microclimate monitoring of historical buildings have recorded time series of either T or RH by means of autonomous data loggers [2,14,26,27,28,29,30,31,32,33] or wireless monitoring systems [1,34]. Some of these research works [2,14,32] have been carried out in European churches to investigate the possible consequences derived from traditional heating in order to improve indoor air conditions for preserving the cultural heritage. Sensors are often located in the historical buildings at a similar distance to the floor level. Regarding the statistical methodology, Principal Component Analysis (PCA) was applied to time series of RH recorded at the Cathedral of Valencia aimed at obtaining clusters of sensors [29,30]. Using the same data set, a novel methodology was recently proposed for classifying the different time series of RH [28]; it was based on sparse Partial Least Squares Discriminant Analysis (sPLS-DA) [35] using input variables extracted from either Autoregressive Integrated Moving Average models (ARIMA), the Holt-Winters method, or functions applied to time series of RH. In a subsequent research, the aforementioned approach was extended using new variables from the Holt-Winters method and Wold decomposition, which was applied to time series of T recorded at the archaeological site of L’Almoina in Valencia [33].

### 1.2. Microclimatic Studies with Sensors Located at Different Heights

The European Friendly-Heating Project [36] highlights the problems caused by installing heating systems in old worship places [14]. In the Mediterranean region, the heating demand in churches is much lower in winter compared with places in Northern Europe, while the requirements for dehumidifying and cooling are greater in spring and summer because of the outdoor humidity and high temperatures [14].

Merello et al. [31] studied time series of T and RH recorded from dataloggers in specific wall orientations and at different levels (floor vs. upper position) at Ariadne’s house in Pompeii (Italy). They applied Analysis of Variance (ANOVA) to either estimates of mean, minimum, or maximum of daily time series of RH and T in order to study the effect of height and wall orientation where the sensors were located on. Likewise, Aste et al. [9] estimated the vertical gradients of T and RH on the entire volume of the Duomo Cathedral (Milan, Italy). They computed the gradient as the difference of T or RH from the lowest sensors compared with those located at the highest levels. Measurements were recorded at 5, 10, 15, and 20 m, from the floor level. Where the maximum height was 45 m, two further measurements were recorded at 35 and 40 m. They found that the gradients in different points were not relevant except for the areas near the entrance to the North aisle, which undergoes higher changes because the gate is used as a primary entrance by churchgoers. Klein et al. [34] installed a wireless monitoring system at The Cloisters, the medieval branch of the New York Metropolitan Museum of Art, in order to improve long-term microclimate monitoring. Sensors were located at different heights in the galleries (e.g., in the Late Gothic Hall the sensor placement height ranged from 0.5 m up to 11.0 m). They evaluated air moisture levels, the thermal stratification along the height of one gallery, and slight temperature gradients between different galleries. They found higher variations in the Hall at the upper level. Using sensors located in different positions and heights, García-Diego et al. [37] applied ANOVA and contour plots to study the performance of the mean T and RH when the heating system was switched on in order to quantify the effects of the heating system on temperature and RH.

Recently, in contrast to traditional technology, Adán et al. [12] used three-dimensional thermal computer vision-based technologies (3D-TCV) for monitoring climatic conditions. This novel approach records dense thermal information in a 3D space, resulting in a data matrix containing 3D coordinates with the associated T and time when the values were recorded. This methodology, combined with traditional recordings of T and RH using a wireless monitoring system, was recently applied at the church of Santos Juanes in Valencia [12]. Data were recorded by the wireless sensors at the lower zone of the principal nave of this church and at the upper zone near the domes. Furthermore, the local surface temperature monitoring system obtained data from three different zones. Such information was studied by computing the standard deviation of surface T. The datasets from 3D-TCV were analyzed by means of thermal orthoimages at different times and graphs of thermal evolution over time [12].

In total, the present research analyzed 21 hourly time series of T from wireless nodes, for seven months during 2017–2018. Sensors were positioned at different heights, ranging from 2 to 13 m from the floor level in the church of Saint Thomas and Saint Philip Neri in Valencia (Spain). The microclimate monitoring system was developed a few years ago as a test prototype [1].

Different monitoring campaigns for the preventive conservation of cultural heritage have been carried out [38,39]. Some of them used autonomous data-loggers, e.g., Hobo data-loggers [40] were employed by Visco et al. [41], data-loggers DS1922L [42] by Valero et al. [43], and DS1923 [44] by Merello et al. [45,46]. There are also studies about microclimates in cultural heritage based on a wired sensor network, composed of different nodes wired to a single microcontroller [29,45]. A more versatile wired/wireless system [47] can be used to solve the problem when using data-loggers, which requires recordings from them to be downloaded manually. The IoT wireless system employed in this study was developed by Perles et al. [1].

The study of thermal conditions in a building can be approached in different ways; for instance, by analyzing the rate of T changes every two height levels (low to high), or by analyzing changes in the characteristics of time series of T at different height levels in the buildings. The latter approach would correspond to classifying time series of T in different clusters according to the height levels. In the first case, a pronounced rate of T change per height might imply phenomena of dust deposition on the walls and artworks in the building, thus it would be necessary to take corrective actions to reduce risks on works of art. In the second case, classifying time series according to different heights (e.g., high, medium and low levels) could be helpful for monitoring the microclimatic conditions in the building. Possible reasons for classifying a set of sensors incorrectly might be the malfunctioning of sensors, changes of thermal conditions where the sensors are located, and the classification method performance, which is influenced by the total number of sensors or the number of sensors per clusters. Thus, sensors incorrectly classified should be evaluated to identify possible setbacks for the artworks.

In order to study vertical temperature gradients and to characterize the time series of T per different height level, two methodologies are proposed. The first one is helpful to determine the existence of a vertical gradient, to estimate the gradient, and to establish the period in a year when such gradient is apparent. This methodology is based on Pearson’s correlation coefficient [48] and linear regression [49]. The second methodology could be used for characterizing the temperature at high, medium and low altitude heights and to determine the main variables that help establish the changes of temperature by level. This methodology that classifies time series is based on sparse Partial Least Squares regression (sPLS) and Linear Discriminant Analysis (LDA). They are employed for classifying purposes, using features from time series as input, which are computed with two methods. The first one corresponds to using some traditional time series functions (i.e., Auto Correlation Function ACF, Partial Auto Correlation Function PACF, periodogram, Moving Range MR), and features defined using quantiles [50]. The second corresponds to using the Holt-Winters method. Finally, with the goal of proposing a plan for long-term monitoring in the church of Saint Tomas and Saint Philip Neri in Valencia, Spain, the thermal condition in this building was analyzed by using both methodologies.

With respect to the first methodology proposed, the temperature gradient has not received much attention yet in the context of art conservation. Some studies have approached the temperature analysis by computing the variation in temperature at different levels of heights [9,34], by using contour graphs of temperatures or by comparing estimates of parameters such as the maximum and minimum temperature [31,37].

Regarding the second methodology proposed, which is used here for classifying time series of T according to different levels of height, it is considered as a novel approach in the context of clustering of time series and cultural heritage. The methodology consists of applying both sPLS [51] with LDA, using features extracted from time series as input. The dissimilarity measures calculated for the method were computed according to other approaches employed in the field of clustering of time series (i.e., profiles of time series, dynamic structure of series, assuming specific underlying models, future forecasts, among others) [52,53,54]. In this case, the dissimilarity measure (i.e., Mahalanobis or Euclidean distance) was computed using a linear combination of a set of variables. These variables could correspond to different approaches, e.g., assuming specific underlying models and future forecasts, profiles of time series and the dynamic structure of series. In this sense, Elorrieta et al. [55] proposed using several features from the field of astronomy and two features that they designed as input for different classification methods (e.g., logistic regression, CART algorithm, boosting, random forest, support vector machine, artificial neural network, and Lasso regression). Some features were extracted from raw data, while others after fitting a harmonic model [55,56]. Concerning the classification algorithms and the methods for computing features from series that are proposed in this paper, this is probably the first time that the combination of both algorithms and such methods are used for classifying and clustering of time series. On the other hand, for art conservation, classifying time series has rarely been explored and it has only been analyzed using PCA [29,30] or sPLS-DA [28,33].

Finally, this research reports a statistical analysis conducted in the church of Saint Tomas and Saint Philip Neri for the first time, which is of relevant interest since inappropriate conditions of temperature can affect the artworks inside the church. Furthermore, the results found in this study might provide guidelines for establishing a plan for thermal monitoring and preventive conservation in similar churches.

The structure of this paper is as follows. Firstly, Section 2 describes the monitoring system, the data set, installation of wireless nodes, as well as criteria for determining the stages of the time series of T, methods for computing features from the series, and the regression method for relating temperature values according to sensor height. The most relevant results and discussion of the different analyses are presented in Section 3. Finally, conclusions can be found in Section 4.

## 2. Materials and Methods

### 2.1. Description of the Monitoring System

The monitoring system used in this paper is the same as in [1], where the details and descriptions of the general system and their components are provided. Furthermore, the reason why the general system and their different components were used is explained.

**Figure 2:**
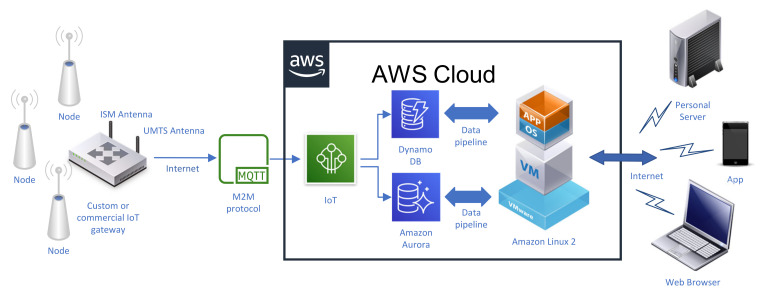
Scheme of the wireless microclimate monitoring system. With respect to the notation: IoT is Internet of Things, ISM is industrial, scientific, medical band, UTMS is Universal Mobile Telecommunications System, and AWS is Amazon Web Services.

The system (Figure 2) was specifically designed for the monitoring needs of cultural heritage buildings and objects. It consists of low-energy wireless sensors, a gateway for collecting the data sampled by the sensors, and a cloud computing infrastructure for data storage, processing and visualization.

**Figure 3:**
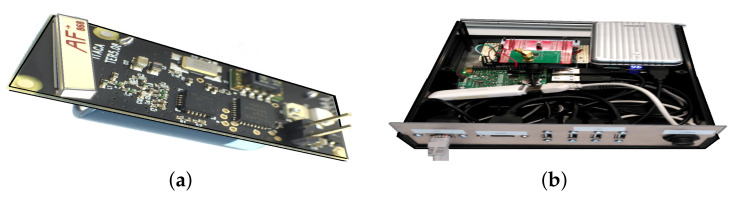
(**a**) Wireless sensor node (approximate dimensions: 4.1×1.5×1.5 cm); (**b**) sink gateway.

A total amount of 21 wireless sensor nodes were installed at the church Saint Thomas and Saint Philip Neri (Figure 3a) for monitoring indoor air conditions. These sensor nodes are built around an ultra-low power C8051F920 microcontroller (Silabs, San José, CA, USA), a CC1101 radio-modem (Texas Instruments, Dallas, TX, USA), a high-density 3.6 V, 1 Ah Lithium-thionyl battery, and a SHT15 chip. The latter is a surface mountable device with an RH sensor and a temperature sensor (Sensirion, Staefa ZH, Switzerland). This device was individually calibrated by the manufacturer (Sensirion). The calibration coefficients are programmed into an inside memory on the chip. To improve the accuracy, these coefficients and the internal voltage regulator are used to calibrate the transmitted signals from the sensors. The accuracy of the SHT15 sensor is $\pm 0.3{\;}^{\circ }$C in the range 10–40 ${}^{\circ }$C [57].

The sensor nodes were used to sample environmental variables of interest, which were transmitted using GFSK (Gaussian Frequency Shift Keying) modulation in the 868 MHz European unlicensed industrial, scientific, medical (ISM) band. All sensors transmit blindly on the same channel without acknowledgment messages from the gateway. This approach allows being very energy efficient at the cost of losing some transmissions.

This sensor node is an adaptation of a previous one devoted to the detection of xylophagous [58] and copes adequately with the requirements of life-span and long distances and thick walls of historical buildings [1].

The gateway, shown in Figure 3b, was built to be as flexible as possible in order to experiment with different approaches, so it was decided to implement it around a Raspberry Pi 3 board (Broadcom Inc., San Jose, CA, USA) and the Linux operating systems. To this base system, we added suitable hardware to support the functionality: a CC1101 radio module and an STM32L04 microcontroller (StMicroelectronics n.v, Geneva, Switzerland) to receive the transmissions of the sensor nodes, a 3G USB dongle to provide mobile connectivity to Internet, and, considering that the gateway is connected to the mains power, a rechargeable lithium-ion battery to provide energy to the gateway during power outages. The main task of the gateway is to collect wireless transmissions of the sensor nodes, store them temporarily in a local database and transmit it to the Internet when connectivity is available. The data transfer is implemented using the MQTT [59] client server publish/subscribe messaging transport protocol.

For the implementation of the cloud infrastructure, it was decided to choose the offering from Amazon Web Services (AWS). The MQTT messages are processed by the AWS IoT cloud service in order to split the message in sensed magnitudes such as temperature, humidity or light level (humidity and light not used in this work), as well as in communication-related parameters (e.g., received signal strength indicator, battery level and message counter). These two types of data flows are stored in a NoSQL (stands for “non SQL” for ones and for “not only SQL” for others) AWS NoSQL DynamoDB database and in an SQL AWS AuroraDB, respectively. In order to allow data access through web browsers, a Linux virtual machine was deployed in the AWS EC2 service, which runs a Redash [60] data visualization dashboard. For statistical analysis, all data collected along the monitored period could be downloaded locally using the AWS Datapipelines service.

Among the advantages of this monitoring system are (1) it is capable of storing an unlimited volume of data, which cloud helps to increase sample frequency and means that updating the recorded information can be carried out every time period, as required (i.e., every second, minute, and another time period), (2) the fact that updating the recorded information does not need to be carried out manually.

The cost of these devices is highly dependent on the type of work to be performed. Mass-market devices tend to be cheaper due to the scale of production, which reduces the cost of the bill of materials and dilutes the engineering cost. In the field of cultural heritage specific devices, and in general in the scientific field, this scale does not apply, so engineering costs are easy to estimate in the cost of the devices and other important costs, such as installation costs (e.g., a wired installation is often very expensive) or personnel costs (e.g., a classical data logger will require periodic battery replacement and manual data downloading), which have to be taken into account. In this particular project, wireless sensor nodes were the best fit in terms of simplicity of installation and personnel requirements, but in a different situation, other options might be more suitable [1].

### 2.2. Experiment for the Calibration of Temperature Sensors

As stated above, the temperature sensor used (SHT15) provides an accuracy of $\pm 0.3{\;}^{\circ }$C according to the manufacturer. In order to obtain the best performance of the deployment, all the sensors were calibrated by comparison before being installed in the church, aimed at estimating their bias and improving the accuracy.

Basically, the set of nodes was located together inside a climate chamber of 23 m${}^{3}$ that was driven by an air cooler in the ceiling (Küba Comfort DP model DPB034). The temperature was controlled inside the chamber during a period of three hours, increasing from $26{\;}^{\circ }$C up to $30{\;}^{\circ }$C. Sensors collected the temperature at a rate higher than a sample per minute.

By computing the mean temperature recorded in the hot stage of the calibration experiment for each sensor, it was found that node M was the one closest to the overall sample mean. Hence, this sensor was regarded as a reference (i.e., with a null bias). Then, for each sensor, the bias was computed as the difference between the mean T recorded by that node, during the hot stage, and the mean T of this reference node (see Table 1).

An independent accurate sensor with a certified calibration would lead to a better estimation of the bias, but, unfortunately, such sensor was not available.

This approach is good enough for the purpose of the present study because the main goal is to analyze the relationship between temperature and the height of nodes, and knowing the real bias per node is of little interest to this paper. Bias values range from −0.28 to +0.28, which is consistent with the accuracy of $\pm 0.3{\;}^{\circ }$C indicated by the sensor manufacturer.

Each value of temperature registered per node during the microclimate monitoring experiment was corrected by subtracting its corresponding bias.

The calibration “in situ” of sensors [41,61] is an effective technique that consists of putting together all node sensors along with a calibrated sensor inside the building that is being monitored. Thus, it is possible to have a climatic condition reference from the calibrated sensor for comparing the records from all nodes. In this study, calibration “in situ” was not considered because, for a massive campaign, the application of this technique requires a greater investment due to the cost of using a calibrated sensor and more time for its implementation. Furthermore, the experiment calibration approach of T used here was possible given that the goal was to compute the differences of T from sensors, instead of estimating the mean of T.

### 2.3. Installation of Wireless Nodes

**Figure 4:**
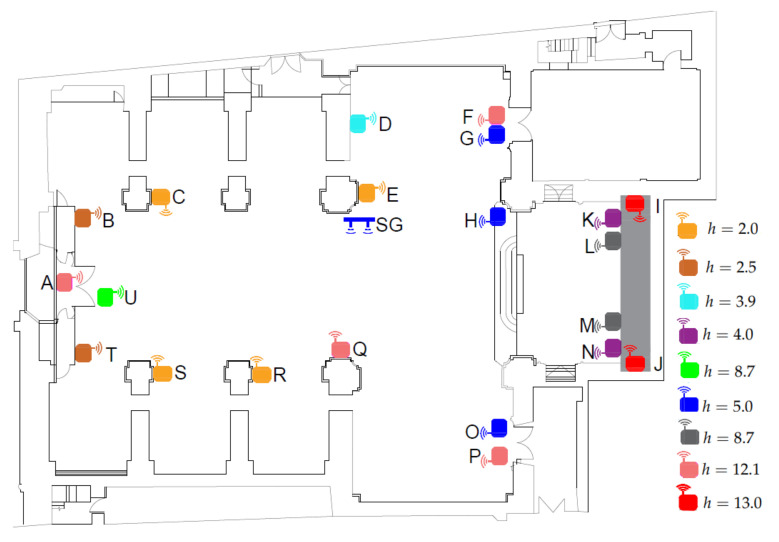
Position of the 21 wireless nodes located in the church of Saint Thomas and Saint Philip Neri (Valencia, Spain). Color refers to height (*h*) of the node (in meters, m). SG indicates the position of the sink gateway that receives data wirelessly from the sensor nodes. The light gray rectangle indicates the position of the main altarpiece (retable).

After the calibration experiment, the 21 wireless sensor nodes were located in the church at different heights (*h*): 2, 2.5, 3.9, 4.0, 4.3, 5.0, 8.7, 12.1, and 13 m from the floor level (Figure 4). The sensors located at each different height are the following:

- h=13.0: nodes I and J were located at the upper part of the retable decorating the presbytery.
- h=12.1: nodes A, F, P, and Q were placed at the upper position, close to the ceiling vaults.
- h=8.7: nodes L and M were also located at the retable.
- h=5.0: nodes G, H and O were placed near to the main altar.
- h=4.3: it corresponds to node U, which was located near the main entrance.
- h=4.0: nodes K and N were also installed at the retable.
- h=3.9: node D was located close to the altarpiece of Saint Joseph.
- h=2.5: nodes B and T were positioned near to the main entrance.
- h=2.0: nodes C, E, R, and S were located, as indicated in Figure 4, at the lowest level.

Some criteria for establishing the position of nodes were the following: (i) to spread out the sensors in different places of the church, (ii) to locate some nodes close to the main entrance and other openings allowing air exchange from outside, and (iii) to install at least 2 nodes at a comparable height for comparison purposes. Moreover, sensors were not placed too close to the floor level because they might be stolen or manipulated by churchgoers. The ideal scenario would have been to spread out the 21 nodes randomly inside the church. However, restrictions such as the building characteristics, the maximum number of nodes available, and the need to prevent problems caused by the movement of people, among other factors, made it impossible to achieve a random distribution of the nodes.

### 2.4. Data Pretreatment

The experiment of microclimate monitoring was carried out from the 1st of August 2017 until the 28th of February 2018 (7 months, 212 days). When programming the communication of sensor nodes with the sink gateway, the time between two consecutive measurements of T $\left(t_{j},t_{j+1}\right)$ was established as a random variable following an exponential distribution with a mean of one hour. The fact that the church has an unheated/natural microclimate indoors better explains the selection of the sampling time of 1 h, which in the case of a heated microclimate, could not be sufficient. The main reason for using this type of distribution was to decrease the probability of data transmission collisions. However, as a drawback, it leads to missing values, which becomes a problem for the methodology of the time series analysis applied here.

Regarding the missing data resulting from the exponential distribution used for establishing two consecutive measurements of T, it was checked that the percentage of missing values per node was approximately the same for the different nodes. By contrast, when the reason was problems of wireless communication with the gateway or electrical failures, the percentage of missing values was greater. In particular, such amount was the highest for node R (41.4%). Taking into account that this node was at 12.5 m from the gateway, which is not too far away, the problem of wireless communication with the gateway was discarded as a reason for having such significant amount of missing values, and the main cause could be a flaw in the electronics. The target was to have a common number of observations per sensor, particularly, one value per hour. For this purpose, all missing values were imputed. Taking into account that the distribution of missing data does not follow any specific pattern (i.e., missing at random [62]), all missing data were imputed using either Stineman interpolation [63] or linear interpolation. The latter was used when the time between two consecutive available measurements of T were less than 2 h (i.e., a single missing value). For the rest of the cases, the Stineman interpolation was used. The interpolation equations were solved for every unknown observation of T between two known values of T. The resulting data were organized as a matrix with 5088 rows (one per hour) by 21 columns (one per node). Finally, each value of temperature registered per node during the microclimate monitoring experiment was corrected by subtracting its corresponding bias. Similar studies have also applied interpolation procedures for the imputation of missing values. Klein et al. [34] estimated temperature and air moisture values using a smooth bivariate interpolant to the scattered sensor data, which is an effective method when the temperature is smoothly varying over short distances. However, this approach most likely loses accuracy near air inlets and outlets in galleries. Trying to overcome this drawback, the authors [34] also applied physics-based models incorporating Computational Fluid Dynamics by prescribing thermal boundary conditions.

### 2.5. Statistical Methods

The methodology is composed of five main steps. First, identification of stages in the time series of T (see Section 2.5.1). Second, estimation of the vertical gradient of T (see Section 2.5.2). Third, computation of parameters from the time series (e.g., sample mean values of Auto Correlation Function ACF, moving range MR, Partial Auto Correlation Function PACF at the first 4 lags, among others, and the additive seasonal Holt-Winters (SH-W) method (see Section 2.5.3). Fourth, analysis of the relationship between T and sensor height, using variables determined in the previous step and sparse Partial Least Squares (sPLS) (see Section 2.5.4). Finally, characterization of temperature at high, medium, and low altitude heights using Linear Discriminant Analysis (LDA) and the latent components from sPLS calculated in the previous step [64]. The R software (version 4.3) was used to carry out the statistical analyses. The main packages used were mixOmics [65], klaR [66], and spls [67].

#### 2.5.1. Identification of Stages in the Time Series

**Figure 5:**
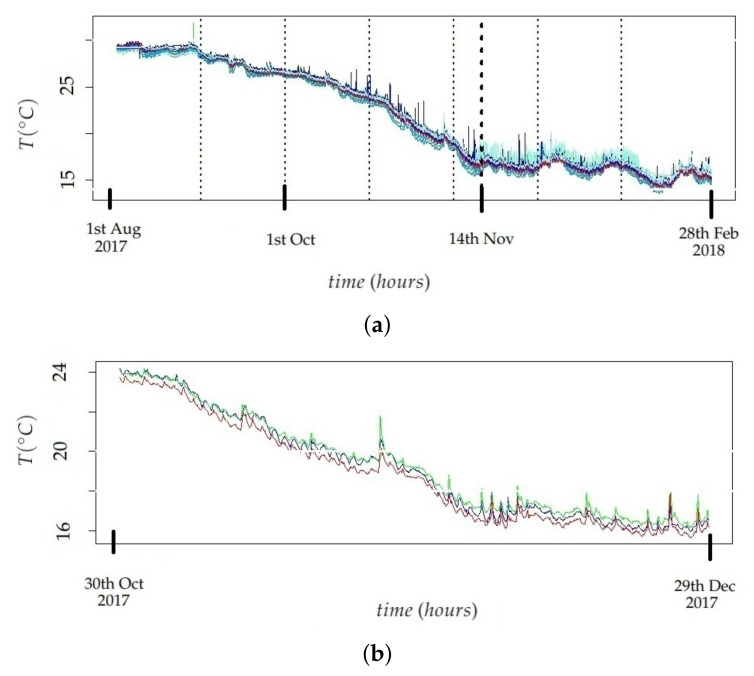
(**a**) Trajectories of temperature for the 21 nodes, before subtracting their corresponding sensor bias. The thick vertical dotted line (November 14th) indicates a change of trend: T slowly decreases before this date on average while, next, the mean T is rather constant. Each thin vertical dotted line separates two consecutive stages (months) that were considered to split the different time series of T. In total, seven stages were considered. (**b**) Trajectories of temperature (before subtracting their corresponding sensor bias) for the nodes B (in blue), H (in green), and M (in red) corresponding to the period between October 30th 2017 and December 29th 2017.

Regarding the monitoring experiment, two main stages were visually identified in the different time series of T: firstly, the average temperature slightly decreases until about November 14th and, next, it becomes approximately stationary (see Figure 5a). By visually inspecting the evolution over time of the time series of T, all of them are quite parallel (see an example in Figure 5b), which can be partly explained by the different position of each node and, moreover, by the bias of each sensor (Figure 5a,b show raw data, prior to the bias correction). The trajectory of M (i.e., the reference node) is depicted in red in Figure 5a,b.

The observed time series of temperature were denoted as T, where $T=(t_{1},\cdots ,t_{j},\cdots ,t_{n})$. By using the supF test [68,69], two potential structural breaks were identified (i.e., changes in the slope of a linear trend), at observation number 763 (September 1st at 6:00 PM, *p*-values< 0.02) and 2797 (November 11th at 12:00 AM, *p*-values< 0.01).

The supF test was applied after calculating the logarithmic transformation and one regular differentiation to the distinct time series. Such logarithmic transformation was employed to stabilize the variance, and the regular differentiation was intended to eliminate the trend of the different time series [70]. The notation employed throughout this article is as follows: r indicates the logarithmic transformation of T, and W refers to one regular differentiation of r. Thus, each value of W corresponds to $w_{j}=r_{j}-r_{j-1}$, where $r_{j}=\ln\left(t_{j}\right)$. The two structural breaks identified lead to splitting the time series into three stages, but this number seems too low for the target of the present work. In order to extract more features from each time series, which presumably might lead to better results, it was decided to split all time series into seven stages, one per month (see Figure 5a). This criterion is consistent with the structural break identified on September 1st, though not with the one found on November 11th, but this issue was considered as a minor drawback.

#### 2.5.2. Estimation of the Vertical Gradient of Temperature for Each Month

With the goal of determining if the vertical gradient is apparent, the Pearson correlation test [48] was applied to different periods of the time series of T (i.e., each month as established in Section 2.5.1). By using the test, it is possible to determine whether the correlation between temperature and height of sensors is statistically significant. Once a period with statistically significant correlation was identified, the slope of the linear relationship was considered as the gradient estimation. Such slope is the derivative of the function that estimates the mean temperature in the month with respect to height.

Consider that the relationship between temperature, $T=(t_{1},\cdots ,t_{n})$, and height, $h=(h_{1},\cdots ,h_{n})$, is determined by the following linear regression model in Equation (1), where, ε=T−E(T|h), E(T|h) is the conditional expectation of T given h, E(ε) is the expectation of the errors ε, and V(ε) is the variance of ε [49]. Details about computing the estimated values and confidence intervals of $\beta_{0}$ and $\beta_{1}$ can be found in [49].

(1) $$\begin{array}{cc} t_{i} & =\beta_{0}+\beta_{1}h_{i}+\varepsilon_{i},\mathrm{where}\\ i & =1,\cdots ,n\\ E(\varepsilon ) & =0\\ V(\varepsilon ) & =\sigma^{2} \end{array}$$

Considering the linear equation that estimates temperatures as a function of height, the derivative of this function is the slope $\beta_{1}$ of the regression line, which is the gradient estimation. The thermal vertical gradient can be interpreted as the rate of increase of T according to height.

In this study, for each month, the gradient was estimated as the slope of the linear regression model by using height (*h*) as the predictor variable and mean temperature as response. This gradient was expressed as ${}^{\circ }$C/m.

The existence of a gradient implies that the correlation between T and *h* is statistically significant, which was checked for each month. If this condition is not fulfilled, there is not enough evidence to affirm that the slope of the regression line is different from zero at the population level. Hence, there is no evidence for a vertical thermal gradient. The proposed method for the calculation of vertical gradients seems reasonable when all sensors are located one above the other, in the same vertical axis, but this is not the case here. However, a preliminary analysis suggested that longitudinal thermal gradients were not relevant in this case, because the ventilation rate of the building is rather limited and because indoor air conditions are not affected by heating or air conditioning systems, which are not installed in this church.

#### 2.5.3. Calculation of Classification Variables

Two methods were used to compute features from the time series, which were applied to the different observed time series (T or W) separately per month. As an exception, each complete time series was also used in the second method, in addition to modeling each month independently. Features will be denoted hereafter as classification variables.

**Figure 6:**
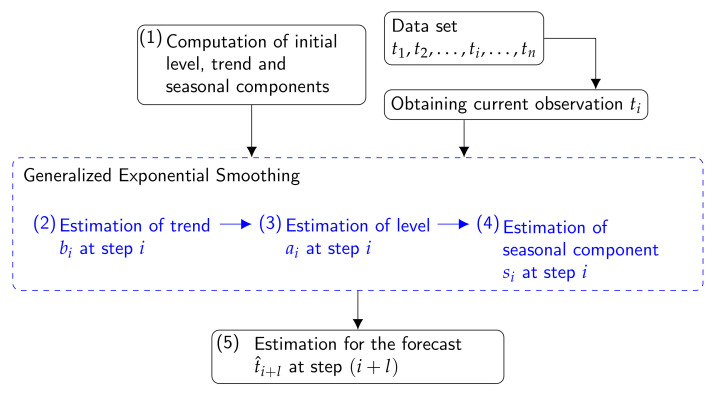
The flow diagram displays five steps for carrying out the additive SH-W method. Step (1) indicates that the initial conditions for the components are computed. Steps (2), (3), and (4), indicate that the slope, level and seasonal component at step *i* are estimated. Finally, step (5) indicates that forecasts (${\hat{t}}_{i+l}$) at step (i+l) are calculated, where l:1,⋯,24.

1. Method 1: Using Time Series Functions
  This method consists of computing features from the observed time series T, in some cases, and from the time series after applying the logarithm transformation and regular differencing to T. The goal of using this transformation and differencing was to stabilize the variance and remove the trend of the series in order to extract information about the seasonal component. Features were calculated by means of values of sample Auto Correlation Function (ACF), sample Partial Auto Correlation Function (PACF), periodogram, Moving Range (MR) [71,72], as well as features defined using quantiles [50]. Each variable was computed for each month and sensor. These correspond to estimates of the following parameters:
  (a) mean.ts: Mean of T recorded in the month. This parameter allows to compare the level of the different time series.
  (b) sd.ts: Standard deviation of T, which provides information about the variability of the recorded values.
  (c) range.ts: Range of T (i.e., by subtracting the minimum to the maximum). It reflects the amplitude of the time series of T and gives information about the dispersion.
  (d) mean.mr: Mean of MR values with order 24 of T. MR computes the moving range for all sequences of 24 consecutive observations.
  (e) median.mr: Median of MR values with order 24 of T. This parameter and the previous one are helpful for capturing the daily variability of the different time series of T.
  (f) mean.acf: Mean of the first 72 lags (l=1,⋯,72) of sample ACF applied to W time series. Each value of ACF for W at lag *l* ($acf_{l}$) is the correlation coefficient between the observations that are lagged for a time gap *l*. It is given by $acf_{l}=cor\left(w_{j},w_{j-l}\right)$, i.e., Pearson’s correlation coefficient between the time series and the lagged values (i.e., the time gap which is considered). The value 72 was used because sample ACF values computed for l=1,2,⋯,72 were comprehended within the limits of a 95% confidence interval in the correlogram. This parameter provides information about the dynamic structure of the time series.
  (g) median.acf: Median of the first 72 lags of sample ACF applied to W. As in the previous case, this parameter can be useful for comparing the dynamic structure of the time series.
  (h) sd.acf: Standard deviation of the first 72 lags of sample ACF of W.
  (i) pacf: First 4 lags (l=1,⋯,4) of sample PACF applied to T. A value of PACF at lag *l* measures the autocorrelation between the observation $t_{j}$ and $t_{j-l}$, which is not accounted for by lags 1 to l−1. The first four values of PACF are usually the most important ones for capturing the most significant autocorrelation information. These four values were computed trying to differentiate the dynamic structure of the different time series.
  (j) maximum.I: Maximum value from the periodogram (I), which is employed for identifying the dominant periods or frequencies of time series of T. This parameter is helpful for recognizing the dominant cyclical behavior in a series.
  (k) range.I: Range of values of the periodogram. This parameter can be useful to compare the impact of the dominant cyclical pattern in the different series.
  (l) maximum.slps: Maximum increase of T in one hour found in the month (i.e., $max\left(t_{j+1}-t_{j}\right)$). This parameter allows the comparison of the maximum changes of T for two consecutive hours, and it is intended to capture the information of abnormal peaks or sudden increases due to occasional events.
  (m) median.abs.sd: Median of absolute values of the deviation between the values of T and the median of T. It is given by median(|T−median(T)|). This parameter is somewhat related to the variance (i.e., average of the squared deviations with respect to the mean) and, hence, it is another measurement of data dispersion.
  (n) t.p.r.m20: It is computed as $\left(T_{60}-T_{40}\right)/\left(T_{95}-T_{5}\right)$, being $T_{a}$ the percentile *a* of values in the month. Thus, it is the ratio of percentiles (60th–40th) over (95th–5th) of T. The numerator is the range of variability corresponding to 20% of the central part of the original time series. The denominator is basically the range of the original time series after removing the lowest 5% and highest 5%. An equivalent interpretation corresponds to the parameters t.p.r.m35, t.p.r.m50, and t.p.r.m80 described next.
  (o) t.p.r.m35: It is computed as $\left(T_{67.5}-T_{32.5}\right)/\left(T_{95}-T_{5}\right)$, which is the ratio of percentiles (67.5th–32.5th) over (95th–5th) of T.
  (p) t.p.r.m50: Ratio of percentiles (75th–25th) over (95th–5th) of T.
  (q) t.p.r.m80: Ratio of percentiles (90th–10th) over (95th–5th) of T.
  (r) p.d.f.p: Ratio of percentiles (95th–5th) over the median of T. This parameter divides the amplitude (range) of the time series, after removing the lowest 5% and highest 5% of observations, by the median of T.
  This list comprises a set of 21 variables that were computed for each one of the seven months, which implies 147 variables in total. They were arranged in a matrix denoted as $X_{1}$ comprised of 21 rows (one per node) and 147 columns (one per variable).
2. Method 2: Additive Seasonal Holt-Winters Method (SH-W)
  This approach calculates features from time series of T, by using the Holt-Winters method (SH-W) [73], which is an extension of the Holt’s method [74]. It captures the level, trend, and seasonality of the different time series and is comprised of the forecast equation and three smoothing equations (i.e., one for the level $a_{i}$, one for the trend or slope $b_{i}$, and one for the seasonal component $s_{i}$) with corresponding smoothing parameters α, β, and γ [75]. According to the additive SH-W, the forecast equation for a time series of T with period length *p* is given by Equation (2) (in this study, *p* is 24), where *k* is the integer part of (l−1)/p, and ${\hat{t}}_{i+l|i}$ is the forecast at step (i+l) [75].
  (2) $$\begin{array}{cc} {\hat{t}}_{i+l|i} & =a_{i}+lb_{i}+s_{i+l-p(k+1)},\mathrm{where}\\ a_{i} & =\alpha \left(t_{i}-s_{i-p}\right)+\left(1-\alpha \right)\left(a_{i-1}+b_{i-1}\right)\\ b_{i} & =\beta \left(a_{i}-a_{i-1}\right)+\left(1-\beta \right)b_{i-1}\\ s_{i} & =\gamma \left(t_{i}-a_{i-1}-b_{i-1}\right)+\left(1-\gamma \right)s_{i-p},\\ \mathrm{where}\;0 & \leq \alpha \leq 1,\;0\leq \beta \leq 1,\;0\leq \gamma \leq 1,\mathrm{and}\;i>s \end{array}$$
  Slope, level and seasonal components at step *i* are estimated by using the three smoothing equations (i.e., for $b_{i}$, $a_{i}$, and $s_{i}$), respectively. If the algorithm converges, *a*, *b* and $s_{1}$ to $s_{p}$ are the estimations for the level, trend or slope and seasonal components. This algorithm was run by using the function HoltWinters of the stats package [76] of R software.
  The flow diagram for the additive SH-W method is displayed in Figure 6. In this diagram, all the steps are repeated with each observation of time series $t_{i}$, i:1,⋯,n. However, in step (1), the initial values of level ($a_{0}$), trend, ($b_{0}$) and seasonal coefficients ($s_{0}$) are only used once to start up the algorithm. The initial conditions are estimated through a simple decomposition in trend and seasonal component by using moving averages. After initialization, steps from (2) to (4) perform the forecast task internally, these values were updated and stored for the next step [76]. In step (2), the estimation of slope requires knowledge of the level at steps *i*, (i−1), and so on until $a_{0}$, as well as slope at steps i−1, and so on until $b_{0}$. In step (3), as in step (2), the equation is solved recursively. Estimation of the level requires knowledge of the level, slope, seasonal components at different steps starting at i−1 (for $a_{i}$), i−1 (for $b_{i}$), i−p (for $s_{i}$), and finishing when the values are $a_{0}$, $b_{0}$, and $s_{0}$. It also requires values of T at steps *i*, and so on until $t_{0}$, where $t_{0}$ is just the oldest data point in the training data set (i.e., a set of observations starting from $t_{1}$ until the current observation $t_{i}$). Note that the weighting coefficients α, β and γ need to be computed for running steps (2), (3) and (4). Such coefficients are calculated by minimizing the squared one-step prediction error [76]. Now that the level, trend and seasonal component at time step *i* have been estimated, the forecast ${\hat{t}}_{\left(i+l\right)}$ at step (i+l) with l=1,⋯,24 can be estimated by using the three values of components together.
  According to this method, the level, trend, and seasonal components are updated over a historical period. For example, when the method is applied per month, the components are updated every hour over each month. If the algorithm converges, *a*, *b* and $s_{1}$ to $s_{24}$ are the estimated values for the level, trend and seasonal components at the last instant of time in the month.
  The level at a time *t* corresponds to a weighted average between the seasonally adjusted temperature and the level forecast, based on the level and slope at the previous instance of time t−1. This component gives an estimate of the local mean (i.e., mean per hour in this study). Regarding the slope component, it expresses the linear increment of the level, over an hour. Finally, the seasonality component estimates the deviation from the local mean, due to seasonality.
  The features calculated per sensor are the following:
  (a) a: Estimated value for the level for each month of the time series.
  (b) b: Estimated value for the trend (slope) for each month.
  (c) s1,s2,…,s24: Estimated values for the seasonal components for each month.
  (d) sse: Sum of squared estimate of errors per month.
  (e) maximum.I: Maximum value of the periodogram computed with the residuals of SH-W for each month.
  (f) mean.acf: Mean of sample ACF of residuals at lags 1 to 72 per month.
  (g) median.acf: Median of sample ACF of residuals at lags 1 to 72 for each month.
  (h) range.acf: Range of sample ACF of residuals at lags 1 to 72 per month.
  (i) Dn: Statistic of the Kolgomorov–Smirnov (KS) normality test [77] of the residuals derived from SH-W, per month of the time series. The KS normality test was employed to compare the empirical distribution function of the residuals with the cumulative distribution function of the normal model.
  (j) Wn: Statistic of the Shapiro–Wilk test (SW) [78] of the residuals per month. This test was used to detect deviations from normality, because of either kurtosis or skewness, or both. The Dn and Wn statistics were also used as classification variables, because they provide information about deviation from normality for the residuals derived from the SH-W method.
  (k) fcast: 24 forecasts of T (i.e., ${\hat{t}}_{i+l|i}$, l=1,⋯,24) for a unique additive SH-W model that was fitted using the complete time series without splitting it in different months.
  Features calculated from (a) to (j) imply a set of 33 variables computed for each month. By including the 24 forecasts as explained in (k), the total number of variables was 33×7+24=255, which were organized as a matrix denoted as $X_{2}$, comprised of 21 rows (one per sensor) and 255 columns (one per variable).

For both data sets, $X_{1}$ and $X_{2}$, those variables with a strongly skewed distribution were transformed with the goal of finding a simple transformation leading to normal distribution. For this purpose, standard (simple) Box-Cox transformations [79] were applied to those variables with a Fisher–Pearson standardized moment coefficient of skewness [80], or with a Fisher coefficient of kurtosis [80] outside the intervals of −2.0 to 2.0. For those variables with a negative skewness, absolute values were used instead of their original ones for applying a Box-Cox transformation. The skewness statistic evaluates the asymmetry of the probability distribution. The kurtosis statistic indicates which variables were heavy-tailed or light-tailed, relative to a normal distribution. Furthermore, the estimates of kurtosis were useful measures for identifying outliers in the different variables.

The percentage of outliers in both data sets was 0.73% in $X_{1}$ and 0.65% in $X_{2}$, which is a small amount. Outliers were discarded, and the resulting missing values were imputed using Non Linear Estimation by Iterative Partial Least Squares (NIPALS [81,82]). Given the low percentage of missing values, their estimation is assumed to be appropriate [83]. Next, once the values were imputed, each column of $X_{1}$ and $X_{2}$ was centered by subtracting its column mean. Furthermore, it was scaled to unitary variance by dividing over its standard deviation.

As both data sets contain more than 100 variables and just 21 rows, a high degree of multicollinearity is expected a priori, which would lead to severely ill-conditioned problems. Furthermore, from a practical point of view, for these high-dimensional data sets, results might be difficult to interpret given the large number of variables. One solution is to extract latent variables that summarize the information using a subset of variables. In this context, many sparse versions [51,84,85,86,87] have been proposed for feature selection purposes. These versions work properly in regression by introducing penalties in the model such as Lasso [88] and Ridge [89].

#### 2.5.4. sPLS

Since Partial Least Squares (PLS) regression was introduced by Wold [81], it has been employed as an alternative approach to Ordinary Least Squares (OLS) regression in ill-conditioned linear regression models that emerge in many disciplines, such as biology, chemistry and economics [87]. PLS is a dimension reduction technique that relates a regressor matrix X and a response matrix Y by computing latent components that correspond to linear combinations of the original variables (predictors). PLS maximizes the covariance between components from two data sets. PLS is computationally fast and the projection of observations on a low-dimensional space allows a graphical representation of observations and variables. Due to these reasons, this method has gained a lot of attention in high-dimensional classification problems [51].

In this study, the data sets $X_{1}$ and $X_{2}$ were analyzed using sPLS with a regression model in an attempt to identify the main variables correlated with sensor height, which will explain the differences in the time series of T according to the distance to the floor level. The information used by sPLS was the following: the response vector, $Y\in R^{n\times 1}$, containing the height of each sensor (n=21), and the regressor matrix, $X\in R^{n\times p}$ ($X_{1}$ or $X_{2}$), which contains the classification variables computed in Section 2.5.3.

sPLS modeled X and Y as a linear regression, where $X=\Xi C+E_{1}$ and $Y=\Xi D+E_{2}=X\beta +E_{2}$, where $\beta \in R^{n\times p}$ is the matrix of regression coefficients, $E_{1}\in R^{n\times p}$ and $E_{2}\in R^{n\times 1}$ are random errors, $\Xi =\left(\xi_{1},\cdots ,\xi_{H}\right)\in R^{n\times H}$ is the matrix of the latent component, where Ξ=XU, with $U\in R^{p\times H}$ as *H* direction vectors, with 1≤H≤min{n,p} and $U=(u_{1},\cdots ,u_{H})$. Furthermore, $\left(u_{h},v_{h}\right)$ is the solution of the optimization problem according to Equation (3) for j=1,⋯,h−1, subject to ${∥u∥}_{2}=1$.

(3) $$\min_{u,v}\{∥M-uv^{⊤}{∥}_{F}^{2}+P_{\lambda_{1}}\left(u\right)\}$$

The optimization problem minimizes the Frobenius norm $∥M-uv^{⊤}{∥}_{F}^{2}=\sum_{i=1}^{n}\sum_{j=1}^{p}{\left(m_{ij}-u_{i}v_{j}\right)}^{2}$, where $M=X^{⊤}Y$, u and v are the loading vectors, and $V=(v_{1},\cdots ,v_{H})$. Furthermore, $P_{\lambda_{1}}\left(u\right)$ is the Lasso penalty function, where $P_{\lambda_{1}}\left(u\right)=\lambda_{1}{∥u∥}_{1}$ [35,86].

This optimization problem is solved based on the PLS algorithm [82] and Singular Value Decomposition (SVD) [90] of a matrix ${\tilde{M}}_{h}$ per dimension *h*. The SVD decomposition of matrix ${\tilde{M}}_{h}$ is subsequently deflated per iteration *h*. This matrix is computed as $U\Delta V^{⊤}$, where U and V are orthonormal matrices, and Δ is a diagonal matrix whose diagonal elements are called the singular values. During the deflation step of PLS, $M_{h}\neq X_{h}^{⊤}Y_{h}$, given that $X_{h}$ and $Y_{h}$ are computed separately, and the new matrix is called ${\tilde{M}}_{h}$. At each step, a new matrix ${\tilde{M}}_{h}=X_{h}^{⊤}Y_{h}$ is calculated and decomposed by SVD. Furthermore, in sPLS algorithm, the soft-thresholding function $g\left(u\right)={\left(|u|-\lambda \right)}_{+}sign\left(u\right)$, with ${\left(x\right)}_{+}=max\left(0,x\right)$, was used in penalizing loading vectors u to perform variable selection in the regressor matrix; thus, $u_{new}=g_{\lambda }\left({\tilde{M}}_{h-1}v_{old}\right)$ [85].

The mixOmics package [91] offers different functions for carrying out multivariate analysis of data sets, with a specific focus on data exploration, dimension reduction and visualization [65]. Among the different functions, it proposes some in order to carry out sPLS. Furthermore, it implements Leave-One-Out cross-validation (LOO-CV) to compare the performance of diverse models with different Lasso penalties. Furthermore, in order to perform variable selection, it employs an algorithm that uses the soft-thresholding function g(u), according to Equation (4). By controlling η instead of the direction vector specific sparsity parameters λ, the method evades combinatorial tuning of the set of sparsity parameters and supplies a bounded range for the sparsity parameter [51].

(4) $$\begin{array}{cc} g(u) & =\left(|u\right|-\eta max_{1\leq j\leq p}|u_{j}|{)}_{+}sign\left(u\right),\mathrm{where}\\ 0 & \leq \eta \leq 1\\ {\left(x\right)}_{+} & =max(0,x) \end{array}$$

The algorithm implemented in the mixOmics package uses the number of variables denoted as keepX for running PLS, instead of the parameter η, while it employs η close to 1. The keepX argument in the package functions is employed in order to evaluate different subsets of variables on each latent component and determine the best number of variables that optimizes the objective function of PLS.

The perf function was used to determine the optimal number of components. The performance of sPLS was evaluated for 10 components using LOO-CV. The optimal number of components was determined by identifying when the further decrease in Root Mean Square Error of Prediction RMSEP is relatively insignificant [92]. RMSEP is defined in Equation (5), where $PRESS_{h}=\sum_{i=1}^{n}{\left(y_{i}-{\hat{y}}_{h(-i)}\right)}^{2}$, with ${\hat{y}}_{h(-i)}$ is the model prediction with 1 to *h* components across all but the *i*-th observation.

(5) $$\begin{array}{c} RMSEP_{h}=\sqrt{\frac{PRESS_{h}}{n}} \end{array}$$

The main criterion for selecting the optimal number of components was RMSEP, while the second one was the goodness-of-fit $R^{2}$ ($0\leq R^{2}\leq 1$). The latter is inflationary and rapidly approaches 1 as the number of model parameters increases. Therefore, it is not sufficient to only have a high $R^{2}$.

In order to determine the optimal number of variables to select on each component, a grid (keepX) of the non-zero elements of the loading vector was assessed on each component, one at a time. The values of three different grids were carefully chosen to achieve a trade-off between resolution and computational time. Firstly, two coarse tuning grids were evaluated before establishing a finer grid. The penalization parameter was chosen by computing the error prediction (RMSEP) with LOO-CV, per component. The tune.spls function was used to determine the optimal number of variables per component. Once the optimal number of components and variables were determined, the final sPLS method was run.

Variable Importance in Projection $VIP_{j}$ [93] was used for computing the overall importance of each predictor variable on the response, cumulatively over the total components. This measure was computed using the loading vectors and the sum of squares per component. Variables with $VIP_{j}>1$ are the most important ones in the regression model.

Although PLS was not originally designed for classification, it has been employed for that objective, with effective performance [51]. With respect to the adjustment of PLS to classification for high-dimensional data, some approaches have been studied, e.g., SPLS Discriminant Analysis (SPLSDA), Sparse Generalized PLS (SGPLS) [87], and sPLS-Discriminant Analysis (sPLS-DA) [35]. Regarding SPLSDA, different variants have been proposed: SPLSDA-LDA (i.e., with linear discriminant analysis) and SPLSDA-LOG, (i.e., with Logistic Regression). These methods aim to improve the PLS classification approaches by using dimension reduction and variable selection simultaneously. In fact, sPLS-DA has been used in order to classify time series in the context of art conservation [28,94].

Likewise, this study proposed a statistical methodology based on SPLSDA [87] for classifying different time series of T in the context of preventive conservation of cultural heritage. SPLSDA computes latent components using sparse partial least squares (SPLS) regression [51]. SPLS selects predictors while reducing dimensions. Next, a classifier is fitted, either Logistic Regression (LOG) or Linear Discriminant Analysis (LDA) [51]. Chung and Keles [51] suggest using a linear classifier because it might be better from an interpretation point of view. The methodology proposed here consists of using sPLS [85] instead SPLS [51]. Once the latent components are computed, LDA is used subsequently.

When examining time series for art conservation, they are generally very similar in distinct positions or height levels of the same building. In this area of research, it is of interest to develop statistical methodologies that can improve the classification of time series with easy interpretation. Such classification can be useful for characterizing and monitoring microclimatic conditions in different zones and heights in a museum, archaeological site or heritage building, with the goal of avoiding problems such as moisture and dust deposition on walls and artworks.

#### 2.5.5. Linear Discriminant Analysis (LDA)

LDA is a supervised method for the discrimination of qualitative variables in which two or more clusters are known a priori and new observations can be classified into one of them, according to their characteristics [89]. In this study, for separating three clusters (K=3) of sensors according to height, LDA was run by using the matrix $X\in R^{n\times d}$, whose elements correspond to values of the *d* components for *n* sensors. The components (d=2) were computed from sPLS (either method 1 or method 2). The clusters that were defined according to the heights (*h*), are the following: 1 (2.0≤h≤4.3), 2 (4.3<h≤8.7), and 3 (8.7<h≤13). The number of nodes per cluster were 10, 5 and 6, respectively. Clusters 2 and 3 comprise of a vertical difference of 4.4 and 4.3 m, respectively, but this value is about half (2.3 m) in cluster 1. This is not an ideal situation, but this criterion was adopted in order to have a similar number of nodes per cluster.

LDA predicts the cluster most appropriate for each of sensor by using Bayes’ theorem, which helps to compute the posterior probability P(y=k|x), for each cluster *k*, k=1,2,3. Suppose that a predictor $X\in R^{d}$ and that the class conditional distribution P(x|y=k) is modeled as a multivariate Gaussian distribution (with mean $\mu_{k}\in R^{d}$ and variance matrix $\Sigma_{k}\in R^{d\times d}$), where all clusters have the same covariance matrix Σ. Then, the log posterior ($\delta_{k}\left(x\right)$) is given by Equation (6), where *D* is the Mahalanobis distance between the data x and the mean $\mu_{k}$. LDA classifies a sensor in the cluster *k*, if the cluster maximizes the log posterior probability $\delta_{k}\left(x\right)$ [89]. Thus, this method classifies a sensor, by accounting for the cluster prior probabilities P(y=k), and the cluster whose mean is the closest to the data x, according to Mahalanobis distance (*D*) [89].

(6) $$\begin{array}{cc} \delta_{k}\left(x\right)= & \log P(y=k|x)\\ \delta_{k}\left(x\right)= & -\frac{1}{2}D+\log P\left(y=k\right)+constant,\mathrm{where}\\ D= & {\left(x-\mu_{k}\right)}^{⊤}\Sigma^{-1}\left(x-\mu_{k}\right) \end{array}$$

Equation (6) can be written as indicated in Equation (7), which implies that this method has a linear decision surface [89].

(7) $$\begin{array}{cc} \delta_{k}\left(x\right)=\log P\left(y=k|x\right)= & \omega_{k0}+\omega_{k}^{⊤}X+constant,\mathrm{where}\\ \omega_{k}=\Sigma^{-1}\mu_{k};\; & \omega_{k0}=-\frac{1}{2}\mu_{k}^{⊤}\Sigma^{-1}\mu_{k}+\log P\left(y=k\right) \end{array}$$

**Figure 7:**
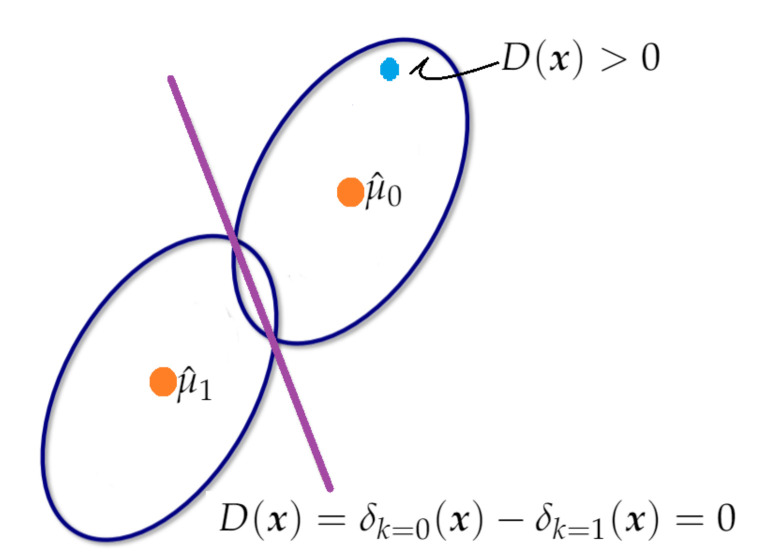
The picture displays two clusters (cluster 0 and cluster 1). If D(x) is greater than 0, the blue point is classified as cluster 0 and otherwise as cluster 1. The purple line corresponds to the boundary of decision, $D\left(x\right)=\delta_{k=0}\left(x\right)-\delta_{k=1}\left(x\right)=0$.

Figure 7 illustrates the boundary of decision, $D\left(x\right)=\delta_{k=0}\left(x\right)-\delta_{k=1}\left(x\right)=0$, for classifying one observation (blue point) from two clusters (K=2). The first cluster has ${\hat{\mu }}_{0}$ as the estimation of the mean, and the second one has ${\hat{\mu }}_{1}$ as the mean. The blue point was classified in cluster 0 because D(x)>0.

LDA can be carried out by first transforming the data in order to have an identity covariance matrix. Next, LDA assigns x to a cluster *k*, taking into account prior probabilities of the cluster and the cluster whose mean is the closest to the observation, according to Euclidean distance [89]. Calculating Euclidean distances in *d*-dimensional space ($\mu_{k}\in R^{d}$) is equivalent to first projecting the data points into an affine subspace of the dimension at a maximum of K−1 [89]. Thus, in this case, LDA determines linear combinations of the components from sPLS for predicting the clusters for the different sensors. This method was run by using the function train (with method="lda") of the caret package [95], and partimat of the klaR package [66] of R software.

In this study, the assumption that each component has a normal distribution for each cluster was verified, as well as whether the variance of the components was the same in all clusters. When the normal condition is not fulfilled, LDA loses accuracy but can still reach a relatively good performance [71]. Results from the methodology proposed (sPLS with LDA) were compared with the results from SPLSDA and sPLS-DA. The classification error rates and number of selected variables from each method were compared. SPLSDA was run by using the function cv.spls of the spls package [67], and sPLS-DA method was run by using the functions perf and tune.splsda of the mixOmics package [65].

## 3. Results and Discussion

**Figure 8:**
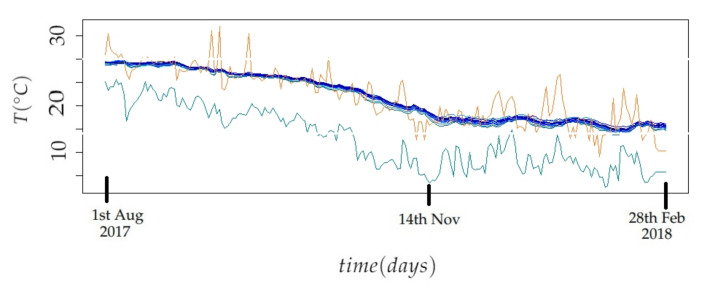
Trajectories of daily-mean temperature over time (days) in the period from August 1st 2017 to February 28th 2018. The green and brown trajectories correspond to the minimum and maximum daily temperature, respectively, in the city of Valencia, Spain. The blue trajectories correspond to temperatures recorded by the 21 sensor nodes inside the church of Saint Thomas and Saint Philip Neri.

The values of T inside the church are influenced by the climatic conditions outside. Figure 8 displays the trajectories of T (days) in the period from August 1st 2017 to February 28th 2018, outside and inside the church of Saint Thomas and Saint Philip Neri. The trajectories of T inside the building correspond to the 21 node sensors employed in this study, while the trajectories of T outside correspond to the minimum and maximum daily temperatures. The trajectories show a similar tendency, as the temperature decreases until November, and T becomes stable after that day. The variability of T, from sensors inside the building is obviously less pronounced than the variability of T outside the church. The values of T inside the church are more influenced by the maximum temperature outside. If the maximum daily temperature is smoothed, it can be observed that the values are quite similar throughout the year to those registered inside the church. This fact is striking, since it would be expected that the temperature inside the temple would be intermediate between the maximum and minimum values of outside air conditions. The main hypothesis is that the maximum outdoor temperature is measured in the shade and under standardized conditions. However, the solar radiation incident on the roof of the church reaches a temperature much higher than that of the surrounding air, which occurs throughout the year because the weather in Valencia is very sunny. This heat is transmitted inside the temple, and would affect the air temperature in the church. A detailed study of heat transmission would be necessary to better study this issue, but it is out of the scope of the present work.

### 3.1. Vertical Gradients of Temperature

**Figure 9:**
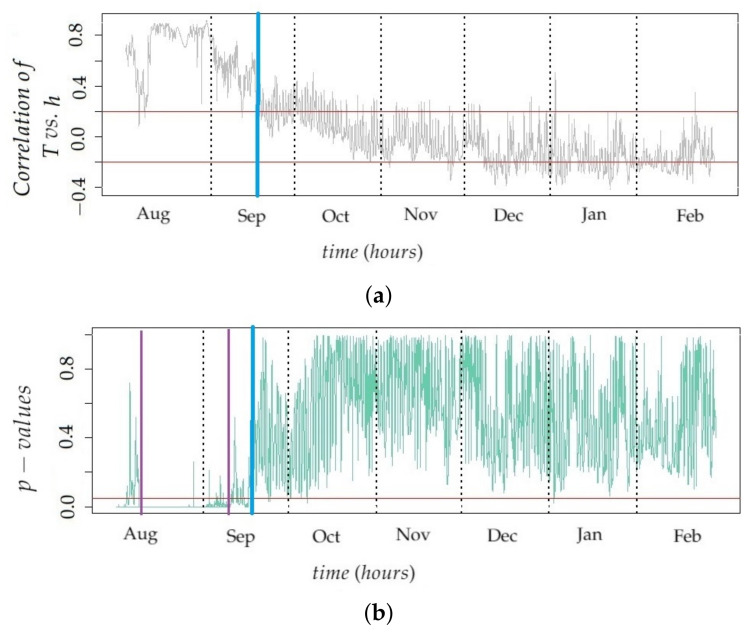
(**a**) Evolution of the correlation coefficient (*r*) between sensor height and temperature, over time (hour). Horizontal red lines correspond to values of −0.20<r<0.20. Dashed vertical lines account for the different months. (**b**) *p*-value from the correlation test over time (hour). The red horizontal line corresponds to a *p*-value = 0.05; for lower values, the correlation was regarded as statistically significant. Purple vertical lines correspond to August 10th at 8:00 AM and September 9th at 11:00 PM. The blue line indicates September 17th at 9:00 PM.

The vertical gradient was estimated for each month by fitting a linear regression model using height and hourly mean temperature as the predictor and response variables, respectively (see Section 2.5.2). The target was to identify in which month the correlation between both variables was statistically significant. It was found that vertical thermal gradients in the church of Saint Thomas and Saint Philip Neri change throughout the year. Figure 9a shows that the correlation between height and hourly values of T is around r=0.8 in August, but it decreases afterward, reaching a null value in October, and the correlation becomes slightly negative in winter. Taking into account that July and August are the hottest months of the year in Valencia, this observed correlation suggests that, in summer, the temperature at the upper part of the central nave is higher than at lower levels. The reason could be the hot temperatures reached during the day in summer in the Mediterranean region [14]. By contrast, in winter, the correlation tends to be slightly negative. However, such correlation is not statistically significant, as described below, which implies that there is not enough evidence to affirm that temperatures in the lower positions tend to be higher in winter. Results reveal that vertical gradients of T are not stable throughout the year, and summer is the only period when vertical airflows might be involved in the phenomenon of dust deposition on walls. For August and September, most values of the correlation coefficient between sensor level and temperature were greater than 0.20 (upper red line in Figure 9a). In fact, the maximum value of r=0.80 was found for August. For the period from August 10th at 8:00 AM to September 9th at 11:00 PM, most *p*-values were less than 0.05 (red line in Figure 9b), which implies that the correlation between height and monthly mean T is statistically significant. Thus, it is possible to establish a linear relationship between them. By contrast, from September 17th, most *p*-values were greater than 0.05 (see Figure 9b). As a consequence, August and September are the most relevant months for explaining the relationship between sensor levels and temperature; in particular, the period from August 10th at 8:00 AM to September 9th at 11:00 PM.

**Figure 10:**
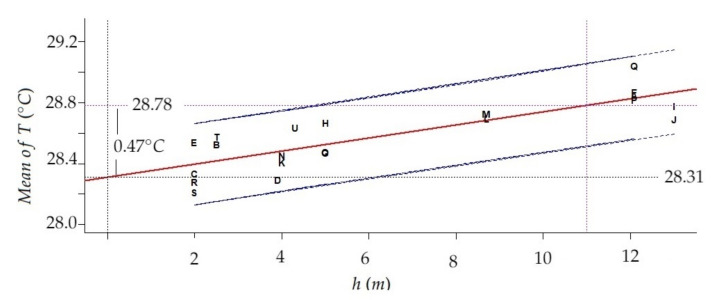
Plot of fitted linear model for mean temperature in the period from August 10th at 8:00 AM to September 9th at 11:00 PM, from each node (codes as in Figure 4) versus height. Prediction limits (in purple) correspond to 95% confidence level. The vertical gradient estimated from 0 to 11 m is about $0.47{\;}^{\circ }$C.

For the period from August 10th at 8:00 AM to September 9th at 11:00 PM, the difference between the mean temperature for the maximum sensor level (13 m) and the minimum sensor level (2 m) was $0.39{\;}^{\circ }$C. Furthermore, estimations of the intercept and slope with their confidence intervals at 95% in the linear regression model using height (predictor variable) and mean of temperatures (response) were 28.31 (28.207, 28.35) and 0.043 (0.030, 0.057), respectively. The coefficient of determination is $R^{2}=70.55\%$. In the period mentioned, at the floor level (height = 0), the estimated mean of T is $28.31{\;}^{\circ }$C. Furthermore, if the height increases by 1 m, the mean of T will increase on average approximately $0.043{\;}^{\circ }$C/m, which implies $0.43{\;}^{\circ }$C per 10 m. This result is consistent with the difference previously calculated. In a vertical difference of 11 m, the model estimates $0.47{\;}^{\circ }$C as the thermal difference. This linear increase can be seen in Figure 10. Given that the gradient corresponds to the slope of the linear regression fitted to the data (mean temperature per node vs. height), it is possible to compare the results from this study with other works reporting differences of temperatures at different height levels.

The vertical thermal gradient quantified here is consistent with a similar study carried out in the Duomo of Milan [9], where the vertical gradient was estimated as $0.033{\;}^{\circ }$C/m. This Cathedral does not have a heating or air conditioning system inside, which would explain the linear gradient and the small variations of T. If the trajectories of T recorded in the Duomo are compared with those from the church of Saint Thomas and Saint Philip Neri, their characteristics are rather similar (e.g., maximum, minimum, trend), probably because the indoor microclimate in both churches is unheated (i.e., natural) without any air conditioning system and the climate in Valencia and Milan is rather similar.

In the Basilica di Santa Maria Maggiore, in Rome, a study of temperature gradients was carried out at heights of 3, 7, and 11 m [96]. A greater vertical gradient was identified in August than in September and December. In August, most time series of temperature underwent an increase by $0.05{\;}^{\circ }$C/m approximately. Regarding the trajectories of T recorded in the church of Santa Maria Maggiore [96], which is relevant for the case of Saint Thomas and Saint Philip Neri, in August and September, higher temperatures were recorded at the maximum height, while the lower ones were found for the minimum height. By contrast, in December, the phenomenon changed, so that lower temperatures were recorded at the maximum height while the opposite occurred near the floor level. The difference between maximum and minimum heights for the sensors were similar in both studies (i.e., 8 and 11 m, for the Basilica in Rome and for the church in Valencia, respectively). Furthermore, the gradient found for August at the Basilica of Santa Maria Maggiore was $0.05{\;}^{\circ }$C/m, which is consistent with the confidence interval of 95%, (0.030,0.057), for the gradient estimated for the period from August 10th to September 9th in the church of Saint Thomas and Saint Philip Neri. However, in other periods such as May, in Santa Maria Maggiore [96], the increment of temperature per meter was at least $0.25{\;}^{\circ }$C/m. The main reason could be the hot air that came in through the front door of the church [96]. According to reported results and the fact that some studies have displayed the effect of temperature gradient on the dust accumulation process under different temperatures [96,97], an important conclusion of both works is that the ventilation of churches can be very important for discussing temperature gradient in height. The ventilation rate should be studied and quantified, as it contributes to the deposition of dust on art works. This issue is discussed in Section 3.2.

Although many studies have analyzed time series of T in the context of art conservation, their focus has not been on comparing parameters (e.g., mean, maximum and minimum) of temperature at different height levels. For example, Merello et al. [31] compared estimation of parameters such as the minimum and maximum of T in distinct positions in a building instead of different height levels of the sensors. Furthermore, they studied the performance of the mean T by using contour plots, which helped to analyze the change of T at different height levels in the building. However, it is not possible to estimate the vertical gradient from this reported study. The methodology proposed by Merello et al. [31] based on ANOVA could be employed to compare the series of T at different levels in the building. However, this method cannot help to discriminate according to the different characteristics of series of T.

In order to study the temperature gradient and identify the best function that explains the changes of temperature according to the height variable (i.e., linear, quadratic or further polynomial orders), it is necessary to employ temperature measures at distinct height levels. In the case of a linear gradient, using a linear regression model seems better than computing the differences between temperatures measured at two levels. The estimation of the model provides a better interpretation of the results. Although linear regression was used in this study, other methods based on smoothing techniques and nonparametric regression [89,98], which relax the usual assumption in several standard models such as the one used here, could be employed. These models are more flexible and they can fit a wide range of structures in the data, e.g., observations from buildings that employed an air conditioning system.

There are several European Standards [15,99,100,101,102,103,104,105,106] for providing guidelines for monitoring, elaboration and study of the microclimatic conditions inside heritage buildings, aimed at art conservation [16,17,18]. According to European Standards EN 15757, variables such as annual average, seasonal variation, short-term fluctuations, and 7th and 93rd percentiles of short-term fluctuations, can be used as reference for specifying the levels of T or RH in order to avoid physical damage in organic and hygroscopic materials. Seasonal variations are computed by using moving average of 30 days, and short-term fluctuations are calculated by using the difference between the instantaneous measures and a moving average [15,107]. Short-term fluctuations are used instead of seasonal cycles because buildings located in cold climates are expected to be equipped with heating systems, which helps to provide more stable seasonal cycles. As a consequence, the indoor conditions are less dependent on the external conditions. However, there is not a complete study on the application of the EN 15757 in all types of climates; thus, it is necessary to assess its methodology in temperate climates and suggested changes, if required [19]. Silva and Henriques [19] carried out a microclimatic study of the Church of St. Christopher in Lisbon (Portugal) with records from November 2011 to August 2013. They analyzed T and RH from 17 thermocouples or portables sensors located in the church in a vertical profile (5 levels: 0.15, 1.50, 3.90, 7.50, and 10 m), in horizontal profiles (4 profiles at different positions), and some surface points of on wall, among others. They studied indoor conditions as indicated by references such as the EN 15757:2010 [15], the Italian National Unification UNI 10829 [108] and the American Society of Heating, Refrigerating and Air-Conditioning Engineers ASHRAE specification [107]. This research is of interest because studies about indoor air conditions in historical buildings in temperate climates are scarce [19]. Although Portugal has a Mediterranean climate, due to its proximity to the Atlantic Ocean, it has a particular climate with winters less cold and summers less warm than climates of other countries in southern Europe. Silva and Henriques [19] define an interval for short-term fluctuations of T of 0.8 ${}^{\circ }$C. This interval or target band was limited by the 7th and 93rd percentiles of T. They suggested following a target band for T in the future as a preventive measure. Furthermore, they found, for example, that the maximum temperature from the sensor at level 3.90 m was 24.9 ${}^{\circ }$C and the temperature minimum was 13.2 ${}^{\circ }$C. Although the trend of the temperature trajectory found for the Church of St. Christopher may coincide with other temperature trajectories for other buildings in Mediterranean countries, the band, minimum and maximum temperatures can be very different. For example, in this study, both the minimum and maximum temperatures were higher than those determined in the Church of St. Christopher. Regarding the previous ideas, it is necessary to evaluate the climatic conditions in buildings located in the Mediterranean climate in order to have reference values for monitoring indoor conditions. Thus, the methodology proposed in the present work for estimating the temperature gradient, could be useful in order to determine reference measures for historical buildings. Furthermore, for buildings located in Mediterranean countries, the confidence interval (95%) of the vertical gradient reported here (0.030 ${}^{\circ }$C/m, 0.057 ${}^{\circ }$C/m) could be considered as a reference measure in summer.

Functions to estimate risk damage in cultural heritage are a permanent subject of study and investigation [109,110]. In [110], a detailed review of such risk damage can be found. However, the quantification of vertical thermal gradients has not received much attention yet regarding the study of risk damage in cultural heritage, though it is well established that T gradients affect dust deposition on walls and works of art [96]. One reason for this can be the difficulty of measuring the speed of the movement of air within the building, which is a consequence of thermal currents dragging particles. Air speed is not easily quantified since it is necessary to model the speed value at each point [111,112,113]. The techniques related to Computational Fluid Dynamics (CFD) analyze physical parameters at each point by using finite elements of volume, mainly T, RH and wind speed. Although these techniques use a computer system for their calculations, they need real measurements to indicate the boundary or input conditions of the problem and, secondly, to verify and validate the results. Therefore, a technique that is capable of quantifying a gradient, such as the one described in the present work, might be useful in a CFD study [111,112,113].

### 3.2. Ventilation of the Church of Saint Thomas and Saint Philip Neri

It has been estimated that the total volume of the church of Saint Thomas and Saint Philip Neri is about 18,000 m${}^{3}$, including the side chapels and the Chapel of the Holy Communion, which is separated from the main nave by a door that always remains open, except in winter. In this chapel there are two tilt-and-turn windows of 1×1.5 m, almost always opened vertically. The Sacrist has ventilation to the outside, but the door that connects the main nave with the sacristy remains closed most of the time. The temple has multiple windows in the upper area, but they do not have openings for ventilation. The main source of ventilation is the large front door, which is rarely fully opened. Ordinarily, the main door gives access to the nartex, which is a wooden structure that serves as a transition between the exterior and interior environment. This nartex has two 2.4×0.9 m doors, which must be pushed to open by the churchgoers. They close automatically by means of springs.

Through the website https://datosclima.es/Aemethistorico/Vientostad.php (accessed on 13 October 2021) it has been found that in Valencia, between August and December 2017, the average wind speed was about 1.4 m/s, which, multiplied by the section of the narthex door (2.2 m${}^{2}$), is equivalent to an average air flow of about 3.08 m${}^{3}$/s. Assuming that during work days this door is open for a total of 200 s (taking into account that the temple can be visited for 6.5 h a day), this equates to an average air volume of 616 m${}^{3}$. Thus, under these conditions, 29 days would be necessary to renew 18,000 m${}^{3}$ of the total volume.

Assuming that on Sundays the attendance of parishioners is much higher, up to perhaps 10 times, it would take about 3 days to renew the total air volume. In any case, these preliminary calculations show that the ventilation rate of the temple is very low. Air renewal rate is an important aspect to consider in the present study. Actually, the fact that the vertical thermal gradient was basically observed in August, could be related to the low ventilation rate during this month. Perhaps a much higher ventilation rate could have homogenized the vertical profile of temperatures and could have altered the results.

### 3.3. Application of sPLS to Identify Key Features Correlated with Height

Next, sPLS was employed to identify the main features from the time series that are correlated with sensor height in the church, which is of interest particularly for those periods where the vertical gradient was not statistically significant. When applying sPLS, as described in Section 2.5.4, according to criteria of RMSEP and $R^{2}$, two components seem to be enough, both when using variables from method 1 and method 2. The total number of selected variables for methods 1 and 2 were 7 and 13, respectively. Variables are sorted in Table 2 by decreasing value of $VIP_{j}$ [93], which was computed for determining the overall importance of each predictor variable on the response, cumulatively over the total components. The values of $VIP_{j}$ for variables in Table 2 are greater than 1, which are the most important ones in the model. Regarding method 1, the variables selected by sPLS correspond to the stages: 1 (mean.ts and mean.mr), 3 (pacf4), 4 (pacf4), 5 (pacf3 and pacf4), and 7 (pacf3). The relevance of pacf3 and pacf4 is difficult to interpret, because these variables imply that the time series are autocorrelated with the values observed 3 or 4 h before. Nevertheless, the most relevant information is the fact that, in August, both mean.ts (mean temperature) and mean.mr (mean moving range with order 24) present nearly the same degree of correlation (r=0.67) with sensor height. Thus, not only the mean temperature tends to be higher at the upper position, but also the daily variability. The reason might be the high temperatures reached in Valencia in August during the day, but they become mild at night.

The most relevant stages were 1 and 4, which correspond to August and November. For both methods, August was the most important month. The mean temperature was important for this month, because the overall mean temperature (mean.ts) was selected for method 1 and, moreover, the local mean at the last instance of time (a, i.e., level) was chosen for method 2. The feature mean.ts was the most important, according to the $VIP_{J}$ for method 1 (see Table 2a) and the level was the 10th variable among the selected ones for method 2 (see Table 2b). Regarding the selected variables from sPLS, mean.ts and mean.mr were the only ones with a statistically significant correlation at α=1% (r=0.86, *p*-value < 0.001 and r=−0.65, *p*-value = 0.001).

In fact, the period from August 10th to September 9th was the most important period for explaining the vertical gradient of temperature. Furthermore, August was the most relevant month for discriminating the temperature according to height. In the same manner, research of the time series of T recorded at the archaeological site of L’Almoina in Valencia found that the most important fluctuations occurred during summer [33], due to the greenhouse effect caused by a skylight that covers part of the ruins. Results reported here are consistent with a similar work that found summer as the most important period for explaining the gradient of T, probably because outdoor temperatures in the Mediterranean region are greater in summer [14].

For method 2, the estimated value for the level (a) was found as relevant in stages 1, 4 and 5. However, the correlation between the level and height was only statistically significant for August (r=0.77, *p*-value < 0.001). In fact, August was the unique month with a pronounced correlation (r=0.78, *p*-value < 0.001) between the level and the mean temperature (mean.ts), which is strongly correlated with the height (r=0.86, *p*-value < 0.001). By taking a look at the coefficients *r* in Table 2b, the highest values corresponds to s6, s7 and s8 (stage 1), which implies a seasonality every 7 h approximately. Furthermore, in this stage, s18 and s19 are relevant.

In simple regression, Y=f(X), so that Y depends on the values of X. In the observed correlation between mean temperature and sensor height, the temperature varies according to the sensor height and, hence, temperature should be regarded as the dependent variable (Y) and height as the predictor (X). In this linear model, the slope can be interpreted as the gradient, as discussed above. Nonetheless, in order to better understand the differences in the time series recorded at the lower vs. the upper positions, multiple linear regression (MLR) was used to fit sensor height (Y) as a function of variables selected from sPLS from method 1. Using these variables in the regression model leads to a high degree of multicollinearity; thus, only two variables were considered in the final model as predictors: mean of T and mean of MR (i.e., moving range of order 24), both from stage 1 (August). The estimation of the height is given by $\hat{y_{i}}=$ 0.36 + 3.24·mean.ts-1.72·mean.mr, i=1,⋯,21. Thus, sensor height can be fitted according to the average temperature in August and a measure of daily variability. The $R^{2}$ for the model was 87%; *p*-values (from F-test and *t*-tests) were less than 0.0001 for determining whether the independent variables in the model are statistically significant. The residual analysis showed that the assumptions of the linear regression model were fulfilled.

**Figure 11:**
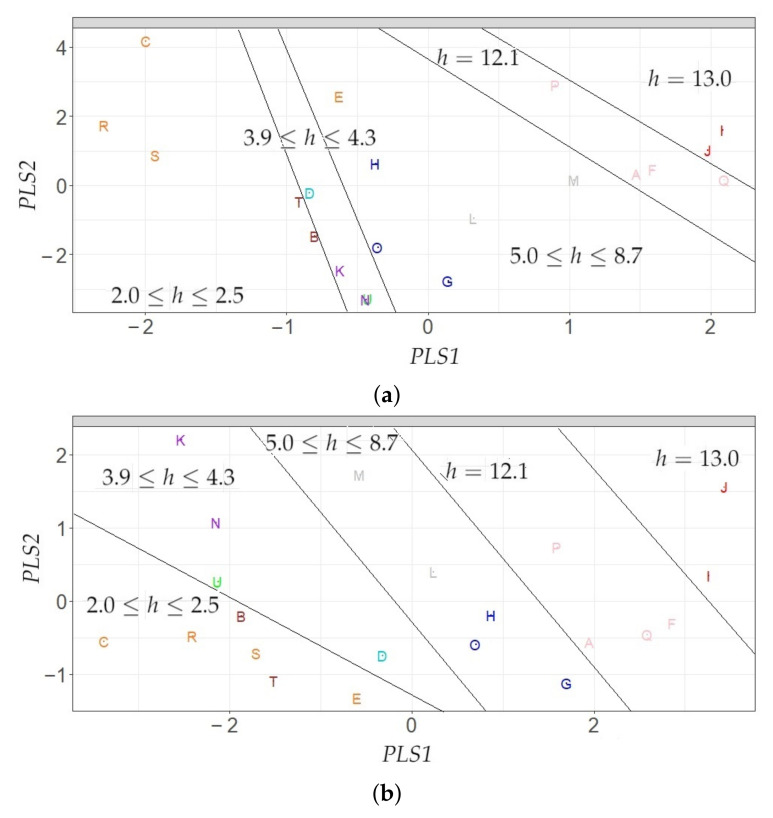
Projection of sensors over the two relevant components (PLS1 and PLS2) on the subspace spanned by the regressor data sets from sPLS; (**a**) using variables from Method 1 and (**b**) Method 2. Sensor codes, represented by letters, as in Figure 4, which were colored according to their height: 13.0 m in red, 12.1 m in pink, 8.7 m in gray, 5.0 m in blue, 4.3 m in green, 4.0 m in purple, 3.9 m in cyan, 2.5 m in brown, and 2.0 m in orange. Solid tilted lines were inserted to better reflect the distribution of nodes in both plots according to height.

Regarding the sPLS results from both methods, Figure 11 shows the projection of sensors over the two relevant components (PLS1 and PLS2) on the subspace spanned by the regressor data sets from sPLS. The projections of sensors were colored according to their height levels (one color per height: 3.0 m in red, 12.1 m in pink, 8.7 m in gray, 5.0 m in blue, 4.3 m in green, 4.0 m in purple, 3.9 m in cyan, 2.5 m in brown, and 2.0 m in orange). According to the tilted solid lines represented in Figure 11, it is possible to establish five classes of sensor nodes according to both methods. It is noteworthy that the solid lines are markedly tilted, which implies that both the first and second components are necessary to achieve a reasonable discrimination of nodes according to height. These classes are adjacent and appear ordered in the plots. Lines in Figure 11a,b separate the different groups, which were denoted as 1 (2.0≤h≤2.5) in blue, 2 (3.9≤h≤4.3) in pink, 3 (5.0≤h≤8.7) in gray, 4 (h=12.1) in purple, and 5 (h=13.0) in green. The tilted solid lines were drawn by visually checking the positions of points, taking into account similar height levels of the sensors. For method 1, node E was classified incorrectly. Nodes T and B are located in the limit of classes 1 and 2, while J appears in the boundary of groups 4 and 5. By contrast, for method 2, all nodes were classified correctly according to the lines drawn in the plot. However, U was located in the limit between class 1 and 2. This classification can be improved by utilizing LDA, which maximizes the differences between the clusters, being the two first components (LDA1 and LDA2) linear combinations of PLS1 and PLS2 components, which in turn are linear combinations of predictor variables from methods 1 and 2. The most important variables per method and component were the following: for method 1, PLS1 was mainly determined by mean.ts and mean.mr, while PLS2 was basically computed by pacf3 and pacf4. For method 2, PLS 1 was determined by the variables a, s6, and s7, while PLS2 was calculated by using s8, s12, s16, s18, s19, s20, and s23. These results suggest that for method 1, the first component explained the level and changes of the levels of the time series of T, while the second explained the autocorrelation of time series. Furthermore, for method 2, the first component explained the level of the last observation of the time series of T, while the second component explained the prediction of the last observation of series at 7, 15, and 24 h past the time of the last observation.

### 3.4. Discrimination of Sensors in Three Categories by Means of LDA

By considering those variables found as relevant from sPLS with two components, LDA was applied in order to check if is possible to discriminate sensors according to their height, and to better understand the variables most relevant for such discrimination. Three categories were established: low, medium, and high elevation.

**Figure 12:**
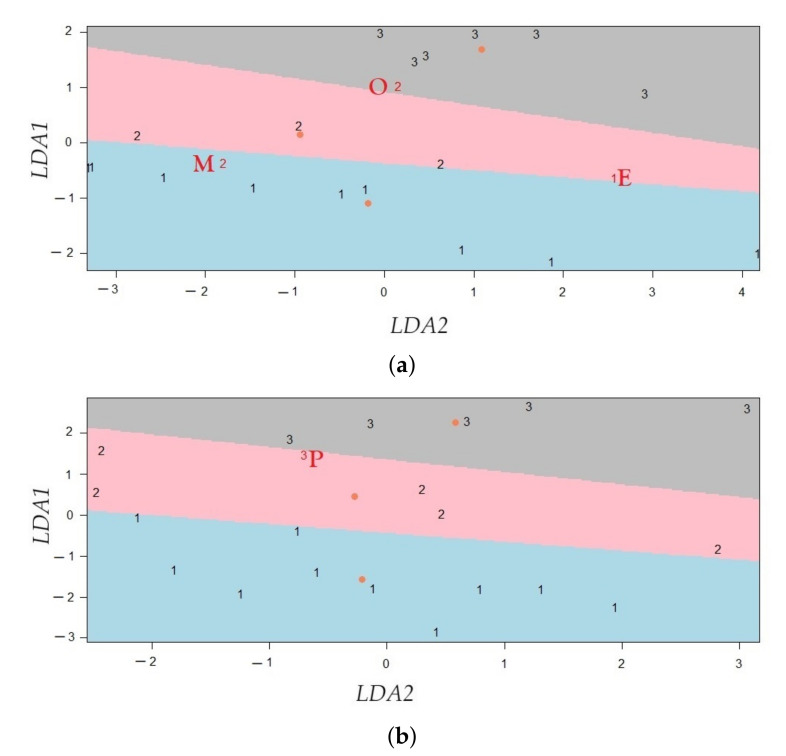
Projection of sensors over the two relevant components (LDA1 and LDA2) from LDA; (**a**) using variables from Method 1, (**b**) Method 2. Three classes were considered according to sensor height (h): class 1 (2.0≤h≤4.3), class 2 (4.3<h≤8.7), and class 3 (8.7<h≤13). Red numbers correspond to sensors wrongly classified.

Figure 12 displays how the sensors are discriminated in three clusters (i.e., blue for cluster 1, red for cluster 2, and gray for cluster 3) by applying LDA. The plot outputs show the projection of sensors over the two relevant components (LDA1 and LDA2). The three lines in the pictures, which separate the three clusters, were determined according to the boundary of decisions from LDA (see Section 2.5.5). By considering variables from method 1, the nodes E, M and O, were incorrectly classified. Nonetheless, nodes E and O are located close the limit of the correct class. Figure 12b shows results from method 2. Only node P was wrongly classified. Although four sensors were classified incorrectly, their projection on LDA1 vs LDA2 appear very close to the boundary of decisions. Therefore, the incorrectly classified sensors do not depart too much from the expected performance.

The discriminant approach used here is based on two steps; firstly, sPLS is applied to identify the most relevant variables and, next, LDA is used for the discrimination. Hence, this procedure was referred to as sPLS with LDA. In order to further discuss the results, two additional discriminant methodologies based on a single step were applied: sPLS-DA and SPLSDA. When comparing the results from the three methods, sPLS with LDA led to the minimum error rates, 14.28% and 4.76% for method 1 and 2, respectively (see Table 3). Using variables from method 1, sPLS-DA selected 10 variables, while sPLS with LDA used 15 (see Table 3a). For method 2, SPLSDA selected 11 variables, less than the other two methods, both of which used 15 (see Table 3b). Computational experiments carried out by Chung and Keles [51] suggested that variable selection performance of SPLSDA improves when the sample sizes increase. However, in the context of art conservation, the number of sensors installed is usually rather small due to restrictions in heritage buildings. Then, a combination of sPLS with LDA can be useful to discriminate the time series according to different levels and zones in this type of building.

For the three classification methods applied, the classification error rates using variables from method 2 were lower or equal to the rates obtained for method 1. In a similar study carried out in Valencia Cathedral [28] and L’Almoina museum [94], sPLS-DA for method 2 obtained the second best results (lower error rate) when comparing with method 1 and other approaches such as ARIMA, ARIMA-GARCH or Wold decomposition. The authors concluded that parameters extracted by applying SH-W has a good performance for a wide range of series [94].

In this study, features defined using quantiles [50] were employed. These variables were not computed in previous studies [28,94] when sPLS-DA was carried out using input features from original time series. Regarding this type of variables, sPLS-DA selected f.p.r.m35 (for stage 5), f.p.r.m80 (for stage 2), and p.d.f.p (for stage 1). Furthermore, SPLSDA selected f.p.r.m20 and f.p.r.m35 (for stages 1, 2, and 5), f.p.r.m50 (for stages 1, 5, and 7), f.p.r.m80 (for stages 1 and 2), as well as p.d.f.p (for stages 1, 2 and 6). However, the classification method based on sPLS with LDA did not select any of these features. In summary, having a variety of time series characteristics can help improve results of the classification of sensors and comparisons of results from different classification methods. Furthermore, finding a common subset of variables was the most important outcome in this case for the classification methods, while other variables improved the results. Different studies have shown efficient results using features from the SH-W method.

sPLS-DA is a one-stage approach that performs, in one step, dimension reduction and selects variables for obtaining the lowest classification error rate. The other methods are two-stage approaches (i.e., SPLSDA, and sPLS with LDA), which only maximize the separation between clusters in the second step. One assumption is that employing two steps instead of one might prevent from obtaining the important variables for classifying the time series. However, the results show that using sPLS with LDA provides the best classification error rates.

Elorrieta et al. [55] proposed a new methodology for classifying time series in the field of astronomy by capturing their peaks. The methodology was based on different classification methods (e.g., Lasso regression, random forest, support vector machine, logistic regression, CART algorithm, boosting, and artificial neural network) and on certain features from the field of astronomy. Furthermore, they proposed two new features to be used as input for the different classification methods [55,56]. The new methodology proposed by Elorrieta et al. [55] shares a common aspect with the approach used here. Both employed a classification algorithm using different characteristics from time series as input. Notwithstanding, the features extracted from time series and classification algorithms are different. Time series from the art conservation field hold different characteristics from the ones in time series from astronomy research. Furthermore, the main goal is to classify stars and results do not need to be interpreted. Nonetheless, some features and all algorithms proposed by Elorrieta et al. [55] can be employed for analyzing the data from art conservation when the aim is to classify time series.

In the field of art conservation, high-dimensional real-world data sets were analyzed to classify time series by using PCA with raw data as input by Zarzo et al. [30] and García-Diego and Zarzo [29]. The relevant principal components were calculated to identify the patterns encoding the highest variance in time series of T and RH. Although PCA does not maximize the separation between clusters for the different time series, it was found that PC1 and PC2 discriminated several clusters from each other, when applying PCA to different time series. Despite the success of PCA in this context, LDA can improve the results because this method maximizes the separation among clusters of time series.

The three methodologies (i.e., sPLS-DA, SPLSDA, and sPLS with LDA) were efficient for classifying time series, as they separate the time series clusters. It should be remarked that these methodologies were numerically stable and competitive in terms of computational efficiency. Consistent results were obtained when the processes were repeated, and the time running was alike and little. In addition, using linear combinations of variables extracted from time series can greatly improve their classification.

In order to study the vertical gradient of T and to characterize the temperature at high, medium and low heights, it would have been more convenient to decide the position of sensors according to a statistical design of experiments considering the same number of sensors at the different levels and different positions in the church. A proper statistical design is important to improve the results and conclusions. However, it is not always possible to use the ideal statistical design because there may be some restrictions in the buildings, such as the characteristics of the building itself, the maximum number of nodes available, and the need to prevent problems derived by the movement of people, among other factors. Nonetheless, results reported here will be helpful for encouraging further studies using an adequate statistical design that can be adapted to these restrictions.

According to the result, the different height levels of sensors can explain the vertical thermal pattern in August. In the other months, the factors that might affect the temperature, are the following: some sensors are located close to windows that were exposed to direct sunlight for a continued period of the day, some sensors are positioned close to halogen lamps reaching a high temperature, among other factors. Work in progress is currently being carried out to study these factors in detail.

## 4. Conclusions

With the goal of proposing a methodology for the multisensor microclimate monitoring in the church of Saint Thomas and Saint Philip Neri (Valencia, Spain), two methodologies were put forward for estimating the vertical gradient of temperature and characterizing the differences between time series at high, medium and low heights.

1. This research reports a microclimatic study in the church of Saint Thomas and Saint Philip Neri in Valencia for the first time, which is of relevant interest because inappropriate conditions of temperature can affect the valuable artworks. The results suggest that temperature gradients in this church were comparable to those estimated at the Duomo in Milan and Santa Maria Maggiore in Rome, Italy. Moreover, it turned out that the identification of such gradients was restricted to a very limited period (August–September) during summertime. Furthermore, the results found in this study might provide guidelines for establishing a plan for thermal monitoring and preventive conservation in similar churches.
2. The first methodology is based on Pearson’s correlation coefficient and linear regression. This methodology, which could help to determine reference thermal gradients for art conservation, could be improved using smoothing techniques and nonparametric regression. Furthermore, taking into account that datasets about indoor air conditions in historical buildings in Mediterranean climates are scarce, the confidence interval (95%) of the vertical gradient found in summer (0.030 ${}^{\circ }$C/m, 0.057 ${}^{\circ }$C/m), could be considered as a reference for further similar studies. Results obtained can be extrapolated to similar scenarios, whether in a heritage building or others, such as an industrial building, warehouse or farm of similar volume and height, with little ventilation, in a similar climate, according to some climate classification criteria (e.g., Köppen [114] and Trewartha [115]).
3. The second methodology proposed here combines sPLS [85] and LDA. Furthermore, it employs variables computed from the seasonal H-W method, or functions that are applied to time series. This methodology helped to obtain parsimonious models with a small subset of variables, leading to satisfactory discrimination and easy interpretation of the different clusters of the time series. Furthermore, it was useful for identifying the most important variables for classifying time series. The variables computed from the seasonal H-W method yielded better results. In other studies, SH-W has also been shown to provide efficient results. This method was more flexible for fitting the distinct time series and obtaining low values of the classification error rate. The new methodology proposed allowed an efficient characterization of T at high, medium and low altitude levels. This approach had the best results according to the classification error rate and number of selected variables, when compared to results from SPLSDA [87] and sPLS-DA [35]. When using variables from seasonal H-W as input for either sPLS with LDA, sPLS-DA, or SPLSDA, both the error rate and the number of selected variables were better.

## Figures and Tables

**Table 1:** Temperature bias (${}^{\circ }$C) per node derived from the calibration experiment.

Node	B	T	U	S	R	C	D	G	E	O	K
Bias	−0.280	0.097	0.160	−0.003	0.069	−0.088	0.077	0.009	−0.019	−0.089	−0.036
**Node**	**N**	**L**	**M**	**I**	**J**	**Q**	**A**	**F**	**P**	**H**	
Bias	−0.046	−0.249	0.000	0.150	0.189	−0.277	−0.098	0.276	0.335	0.175	

**Table 2:** Results from sPLS: variables selected (V), ordered from top to bottom according to $VIP_{j}$. The monthly stage is indicated as Stg1 (August) to Stg7 (February). Correlation coefficients (*r*) of each selected variable vs. sensor height, and the corresponding *p*-values of the correlation test. Results are presented in accordance with the variables used in sPLS: (**a**) Method 1 and (**b**) Method 2.

(a)						(b)		
	Stage	V	*r*	*p*-Value	Stage	V	*r*	*p*-Value
1	Stg1	mean.ts	0.86	0.000	Stg4	a	−0.33	0.138
2	Stg5	pacf4	0.28	0.217	Stg1	s7	0.83	0.000
3	Stg4	pacf4	0.36	0.108	Stg1	s8	0.79	0.000
4	Stg7	pacf3	0.51	0.019	Stg1	s6	0.80	0.000
5	Stg3	pacf4	0.43	0.054	Stg1	s19	−0.77	0.000
6	Stg1	mean.mr	−0.65	0.001	Stg5	a	−0.31	0.178
7	Stg5	pacf3	0.31	0.167	Stg4	s12	0.74	0.000
8					Stg1	s18	−0.75	0.000
9					Stg4	s6	−0.23	0.319
10					Stg1	a	0.77	0.000
11					Stg7	s20	−0.41	0.065
12					Stg7	s16	0.70	0.000
13					Stg4	s23	−0.69	0.001

**Table 3:** Classification error rate and number of selected variables (N) using sPLS-DA, SPLSDA, and sPLS with LDA. Results are presented according to the method used for computing the features from the time series: (**a**) Method 1 and (**b**) Method 2.

(a)			(b)	
Classification Method	Error Rate (%)	N	Error Rate (%)	N
sPLS-DA	35.06	10	18.75	15
SPLSDA	19.04	42	19.04	11
sPLS [85] with LDA	14.28	15	4.76	15

## Data Availability

Data sets will be available at a public repository before publication.
